# An ATX-LPA_6_-Gα_13_-ROCK axis shapes and maintains caudal vein plexus in zebrafish

**DOI:** 10.1016/j.isci.2021.103254

**Published:** 2021-10-12

**Authors:** Ryohei Okasato, Kuniyuki Kano, Ryoji Kise, Asuka Inoue, Shigetomo Fukuhara, Junken Aoki

**Affiliations:** 1 Graduate School of Pharmaceutical Sciences, University of Tokyo, 7-3-1, Hongo, Bunkyo-ku, Tokyo 113-0033, Japan; 2 Graduate School of Pharmaceutical Sciences, Tohoku University, 6-3, Aoba, Aramaki, Aoba-ku, Sendai 980-8578, Japan; 3 AMED-LEAP, Japan Agency for Medical Research and Development, 1-7-1 Otemachi, Chiyoda-ku, Tokyo 100-0004, Japan; 4 Department of Molecular Pathophysiology, Institute of Advanced Medical Sciences, Nippon Medical School, 1-1-5, Sendagi, Bunkyo-ku, Tokyo 113-8602, Japan

**Keywords:** Cell biology, Developmental biology

## Abstract

Lysophosphatidic acid (LPA) is a potential regulator of vascular formation derived from blood. In this study, we utilized zebrafish as a model organism to monitor the blood vessel formation in detail. Zebrafish mutant of ATX, an LPA-producing enzyme, had a defect in the caudal vein plexus (CVP). Pharmacological inhibition of ATX resulted in a fusion of the delicate vessels in the CVP to form large sac-like vessels. Mutant embryos of LPA_6_ receptor and downstream Gα_13_ showed the same phenotype. Administration of OMPT, a stable LPA-analog, induced rapid CVP constriction, which was attenuated significantly in the LPA_6_ mutant. We also found that blood flow-induced CVP formation was dependent on ATX. The present study demonstrated that the ATX-LPA_6_ axis acts cooperatively with blood flow and contributes to the formation and maintenance of the CVP by generating contractive force in endothelial cells.

## Highlights

• Blocking an ATX-LPA_6_-Gα_13_-ROCK axis causes malformation of the caudal vein plexus
• The axis also contributes to maintaining the fine structure of the caudal vein plexus
• Activation of LPA_6_ induces vasoconstriction
• Caudal vein plexus formation evoked by blood flow is dependent on an ATX-LPA_6_ axis

## Graphical abstract

**Figure:**
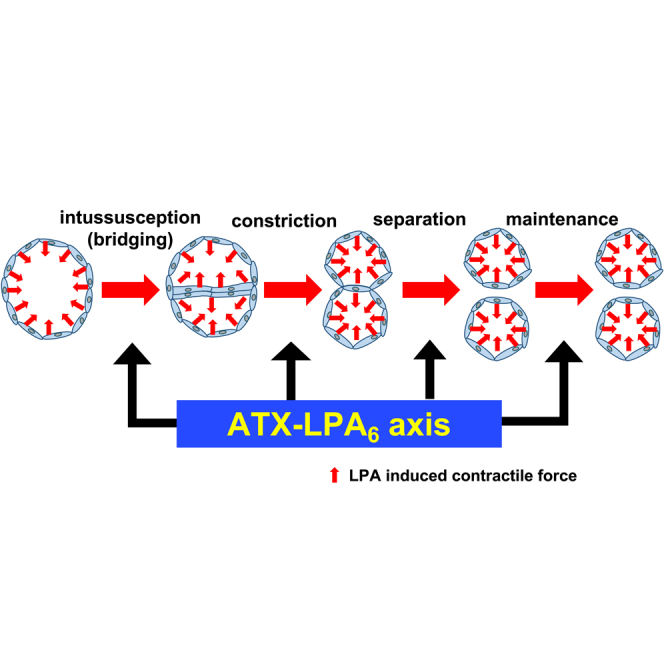

## Introduction

Vascular endothelial growth factor (VEGF, mainly VEGF-A) has received the most attention as the central molecule responsible for angiogenesis (Apte et al., 2019). VEGF-A is upregulated by hypoxia and leads to sprouting angiogenesis in interstitial areas where blood supply is inadequate. After initial vessel structures have been established, the vessels are filled with blood and undergo further vascular remodeling, including stabilization, diameter adjustment, and regression, resulting in a proper vascular network (Jones et al., 2006; Jones, 2011; Tanaka and Laurindo, 2017). Blood flow is particularly important in vessel development after the initial vessels are formed. Blood-derived factors may also have some roles in this process. Among the various blood-derived factors, lysophosphatidic acid (LPA) and sphingosine 1-phosphate (S1P), the two major bioactive lysophospholipids, have been suggested to be potential angiogenic factors. S1P is present in the bloodstream at a high concentration (∼1 μM). It contributes to vascular stabilization by strengthening endothelial cell-cell adhesion via several G protein-coupled receptors (GPCR) specific to S1P (Yanagida and Hla, 2017). LPA has also been implicated in embryonic blood vessel formation because knockout mice of an enzyme involved in LPA synthesis (autotaxin (ATX)) and LPA receptors (LPA_4_ and LPA_6_) showed similar vascular defects in embryos around E10.5 (Tanaka et al., 2006; van Meeteren et al., 2006; Koike et al., 2009; Kano et al., 2019; Yasuda et al., 2019).

The vascular plexus is a fine structure of tubes interconnected with each other and is distributed in many normal tissues, including the brain, liver, and lymph node, and also in pathophysiological conditions (Pearce, 2006; Restrepo et al., 2015; Uhl et al., 2016). Despite its common presence, the molecular mechanisms involved in forming and maintaining the plexus structure are not fully understood. The most analyzed model of the venous plexus is the caudal vein plexus (CVP) in zebrafish (Wiley et al., 2011; Mouillesseaux et al., 2016; Goetz et al., 2014; Xie et al., 2018; Karthik et al., 2018; Nagasawa-Masuda and Terai, 2017; Choi et al., 2011; Wakayama et al., 2015). The CVP transiently develops during embryonic development in zebrafish. During zebrafish development, the caudal vein (CV), which is the source of the CVP, arises near the caudal aorta (CA) by 24 h post fertilization (hpf) (Herbert et al., 2009). The CVP is then formed by the synergistic interaction of sprouting angiogenesis and intussusceptive angiogenesis, in which the sizable primitive CV undergoes morphological changes to form fine and interconnected vessels (Karthik et al., 2018). Several preceding reports indicated that factors affecting the actin filament organization, including blood flow (Goetz et al., 2014; Xie et al., 2018; Karthik et al., 2018), Yap/Ctgf (Nagasawa-Masuda and Terai, 2017), HMG-CoA reductase (activates RhoA by geranylgeranylation) (Choi et al., 2011), and Bmp/Cdc42 (Wakayama et al., 2015) regulated the CVP formation. These studies also suggest that factors that alter the morphology of endothelial cells are involved in CVP formation by regulating the dynamics of actin fibers. Other endothelial cell morphology-altering factors, particularly those that regulate actin fibers, are potential regulators of CVP formation.

ATX is a secreted lysophospholipase D (lysoPLD) that produces a bioactive lipid, lysophosphatidic acid (LPA) from lysophospholipids such as lysophosphatidylcholine (LPC) (Umezu-Goto et al., 2002). LPA produced by ATX, in turn, activates six G protein-coupled receptors (GPCRs, LPA_1-6_) and exerts various pathophysiological roles, including embryo implantation (Ye et al., 2005; Aikawa et al., 2017), development of endometriosis (Kowalczyk-Zieba et al., 2019), fibrosis of lung (Tager et al., 2008; Swaney et al., 2010) and kidney (Sakai et al., 2019), and neuropathy pain (Inoue et al., 2004, 2008). ATX and LPA receptors also have a variety of biological functions during development. LPA_1_ knockout (KO) mice showed impaired brain development (Estivill-Torrus et al., 2008) and chondrogenesis (Nishioka et al., 2016). ATX KO mice showed defects in the formation of vascular systems and died at embryonic day 9.5–10.5 (Tanaka et al., 2006; van Meeteren et al., 2006; Koike et al., 2009). More recently, double knockout mice of Gα_13_-coupling LPA_4_ and LPA_6_ were reported to have a phenotype (embryonic lethality and a defective vascular system) similar to that of ATX KO mice (Kano et al., 2019; Yasuda et al., 2019).

Interestingly, endothelial cell-specific knockout of Gα_13_ led to a similar embryonic lethality in mice (Ruppel et al., 2005). In addition, in cultured endothelial cells (HUVECs), LPA induced dramatic morphological changes by inducing actin stress fiber formation via LPA_6_ and the downstream signaling proteins, Gα_13_, RhoA, and Rho kinase (Yukiura et al., 2015). These observations suggest that LPA produced by ATX has a critical role in the formation of embryonic blood vessels through LPA_4_ and LPA_6_ by regulating endothelial cell shape and that this role is mediated by Gα_13_, RhoA, Rho kinase, and the resulting actin fiber modification. In addition, LPA_4_/LPA_6_ and downstream Gα_13_ signaling have been suggested to positively regulate Yap/Taz transcription factors (Yasuda et al., 2019). These transcription factors induce endothelial cell sprouting, possibly by down-regulating β-catenin and Notch ligand DLL4 (Yasuda et al., 2019). Although LPA is a crucial regulator of actin fibers in endothelial cells, its precise role in angiogenesis through the actin fiber modification is obscure. A detailed *in vivo* analysis of endothelial cell dynamics would provide a clue to understanding the role of LPA signaling. However, the mouse model is not suitable for such studies because of the difficulty of observation and manipulation.

The ATX-LPA receptor axis is well conserved among vertebrates, including zebrafish (Fukushima et al., 2015; Yukiura et al., 2011). In zebrafish, genes encoding LPA receptor (LPA_1_-LPA_6_) and ATX are highly conserved, showing 30 to 90% homology to the corresponding mammalian orthologs. In addition, the biochemical functions of zebrafish LPA receptors and ATX are well conserved (Yukiura et al., 2011). Zebrafish are well suited for developmental studies because of their small size, transparency of embryos, and development outside of the mother (Lawson and Wolfe, 2011; Hogan and Schulte-Merker, 2017). Another advantage of using zebrafish is that transgenic (*Tg*) zebrafish, which make it possible to observe organ development in embryonic stages, are available. For example, fluorescent zebrafish *Tg (fli1:EGFP)* make it possible to monitor blood vessel formation processes in different developmental stages (Isogai et al., 2003).

Morpholino (MO)-based knockdown approaches in zebrafish have shown that ATX-LPA signaling plays essential roles in blood vessel formation (Yukiura et al., 2011), lymphatic vessel formation (Lee et al., 2008), and oligodendrocyte differentiation (Yuelling et al., 2012). However, a recent study comparing the phenotypes of MO-induced gene knockdown embryos and gene mutants revealed the vulnerability of the MO-based tools to dissect the gene functions because of the off-target effects of MO (Kok et al., 2015). Recently, LPA_1_ KO embryos were shown to have abnormalities in chondrogenesis (Nishioka et al., 2016) and LPA_3_ receptor was shown to be involved in megakaryopoiesis (Lin et al., 2018). However, neither mutant showed any abnormalities in angiogenesis.

We previously biochemically characterized zebrafish ATX (ATXa), which at that time was a unique ATX gene in the zebrafish gene database (Yukiura et al., 2011). Accordingly, we generated KO fish of *atxa* using the TALEN system. Surprisingly, the resulting *atxa* mutant developed with an intact vascular system. Notably, ATXa KO zebrafish had plasma ATX activity nearly equal to that of wild-type fish. This finding led to the discovery of a second ATX gene, *atxb*, in zebrafish (Kise et al., 2019). Both *atxa* and *atxb* encoded functional ATX proteins, and an ATX inhibitor ONO-8430506 developed against mammalian ATX efficiently blocked the lysoPLD activity of the two ATX proteins (Kise et al., 2019). In this study, to explore the biological roles of ATX in zebrafish, we generated the *atxb* and *atxa*/*atxb* double zebrafish mutants. As a result, we unexpectedly found that ATXb has a role in forming and maintaining a specific vessel, i.e., caudal vein plexus (CVP)

## Results

### Establishment of *atxb* mutant fish

First, we generated ATXb mutants and ATXa/ATXb double mutants using the genome editing technology CRISPR from the previously established ATXa mutant (Kise et al., 2019). We established two lines of ATXb hetero mutants (*atxb*^*ro1*^ and *atxb*^*ro2*^ alleles) and two lines of ATXa/ATXb double-hetero mutants (*atxb*^*ro1*^ and *atxb*^*ro2*^ alleles) carrying a frameshift mutation in amino acid residues in the vicinity of threonine^195^ of ATXb, which is predicted to be the catalytic center (Yukiura et al., 2011; Kise et al., 2019) (Figure S1). Of note, both mutant ATXb proteins encoded by the two mutant alleles (*atxb*^*ro1*^ and *atxb*^*ro2*^) lack the catalytic center, which is essential for the catalytic activity of the ATX, which shows that the ATXb products produced from the mutant alleles lack catalytic activity. Next, we determined the genotypes of adult fishes obtained by crossing ATXa/ATXb double mutants (*atxa*^−/−^/*atxb*^+/−^ × *atxa*^+/−^/*atxb*^+/−^). The ratio of the resulting six genotypes (*atxa*^+/−^/*atxb*^+/+^, *atxa*^+/−^/*atxb*^+/−^, *atxa*^+/−^/*atxb*^−/−^, *atxa*^−/−^/*atxb*^+/+^, *atxa*^−/−^/*atxb*^+/−^, and *atxa*^−/−^/*atxb*^−/−^) roughly followed the Mendelian law (Figure S2).

Little ATX (lysoPLD) activity was detected in the plasma from ATXa/ATXb DKO adult fishes, whereas a small amount of lysoPLD activity (about 10% of that of wild-type zebrafish) was detected in the plasma of ATXb KO (*atxa*^+/+^/*atxb*^−/−^) fish (Figure S3). These results indicate that ATXb and ATXa are the major and the minor ATXs in zebrafish, respectively. They also suggest that zebrafish do not have a third ATX gene. Thus, we unexpectedly found that loss of ATX was not lethal during development in zebrafish unlike in mice.

### Abnormal caudal vein plexus (CVP) structure in the *atxb* mutant

**Figure 1:**
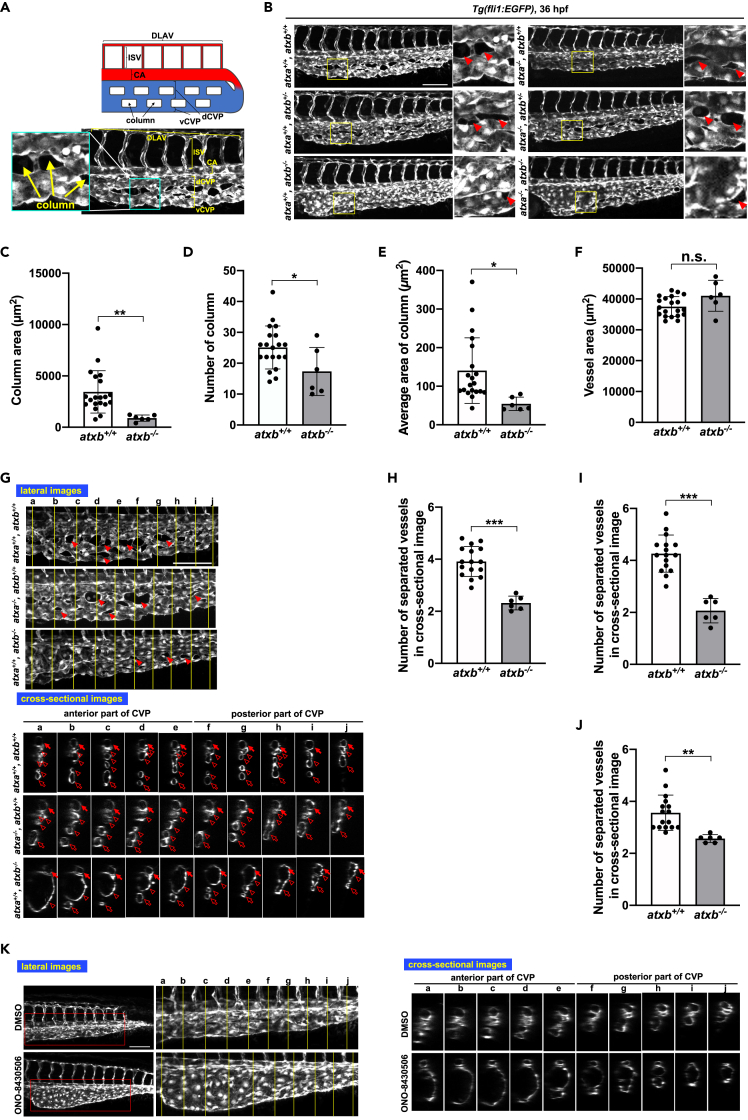
Abnormal caudal vein plexus (CVP) structure in *atxb* mutant embryos (A) Schematic diagram of blood vessels in the caudal region of the zebrafish embryo. DLAV, dorsal longitudinal anastomotic vessel; ISV, intersegmental vessel; CA, caudal aorta; dCVP, dorsal part of CVP; vCVP, ventral part of CVP. (B) Projection view of confocal z stack images of CVP from the lateral side of wild-type (*atxa*^+/+^/*atxb*^+/+^), *atxb* heterozygous (*atxa*^+/+^/*atxb*^+/−^) and homozygous (*atxa*^+/+^/*atxb*^−/−^), *atxa* homozygous (*atxa*^−/−^/*atxb*^+/+^), *atxa* homozygous *atxb* heterozygous (*atxa*^−/−^/*atxb*^+/−^) and *atxa*/*atxb* double-homozygous (*atxa*^−/−^/*atxb*^−/−^) mutant embryos at 36 hpf. Enlarged images of the area surrounded by squares are positioned on the right side. Arrowheads show the column structure formed between vessels. Scale bar, 100 μm. (C–F) Quantitative evaluation of CVP’s morphology from confocal images from the lateral side. Ten somites from the end of the yolk extension were evaluated. Data were shown as mean with SD of sixteen *atxb*^*+/+*^ and six *atxb*^*−/−*^ embryos. p value was calculated by the student’s t test (∗p < 0.05; ∗∗p < 0.01; n.s., no significance). (C) The total area of columns in the ten somites was quantified by Zen 2 (blue edition) software and shown. (D) The total number of columns present across the ten somites. (E) The average area of columns (Total area of columns (C) divided by the number of columns (D)). (F) Total vessel area. The EGFP-positive area was quantified as a vessel area. (G) The cross-sectional single-plane images of CA, dCVP and vCVP from wild-type (*atxb*^+/+^/*atxb*^+/+^), ATXa KO (*atxa*^−/−^/*atxb*^+/+^) and ATXb KO (*atxb*^+/+^/*atxb*^−/−^) embryos at the positions indicated by yellow lines in the upper lateral view are shown in the lower side. Ten somites from the end of the yolk extension were analyzed. Somites a to e and somites f to j were defined as anterior and posterior somites, respectively. Arrowheads indicate the column structures in the lateral images. Arrows, hollow arrowheads and hollow arrows in the cross-sectional images indicate the CA, dCVP and vCVP, respectively. Note that in *atxb*^−/−^ embryos CA and dCVP were not separated in the posterior part. Scale bar, 100 μm. (H–J) Quantitative evaluation of CVP’s morphology from the cross-sectional images. Data were shown as mean with SD of sixteen *atxb*^*+/+*^ and six *atxb*^*−/−*^ embryos. p value was calculated by the student’s t test (∗∗p < 0.01; ∗∗∗p < 0.001). The graphs show the average number of separated vessels in the cross-sectional images from ten somites (somites a to j, H), five anterior somites (somites a to e, I), and five posterior somites (somites f to j, J). (K) Projection views of confocal z stack images from lateral side and cross-sectional images of CVP at 36 hpf. Wild-type embryos were treated with ATX inhibitor, ONO-8430506, from 25 to 36 hpf. Schematic diagrams of the protocol are also shown in the upper side. Scale bar, 100 μm. See also Figures S1, S5, and S6, Videos S1 and S2.

We noticed that at 36 hpf ATXb KO (*atxb*^−/−^) embryos (both *ro1* and *ro2* lines) showed an abnormal blood flow, especially in the caudal part. In ATXb KO embryos, blood cells flowed very slowly through the veins and did not reach the tail (Video S1 and S2). Of note, the heart rates were comparable between wild-type and ATXb KO embryos (Figure S4). To visualize vessels, we crossed the ATXb and ATXa/ATXb mutants with a *Tg* lineage, *Tg(fli1:EGFP)*, in which EGFP is expressed specifically in endothelial cells, and analyzed the process of vessel formation during development. At 36 hpf, vessels in the posterior parts consisted of the dorsal longitudinal anastomotic vessel (DLAV), intersegmental vessel (ISV), caudal aorta (CA), and caudal vein plexus (CVP) from the dorsal to the ventral sides (Figure 1A), as was reported previously (Isogai et al., 2001). At this stage, CVP consists of dorsal (dCVP) and ventral (vCVP) parts (Karthik et al., 2018; Nagasawa-Masuda and Terai, 2017; Wakayama et al., 2015) (Figure 1A). Time-lapse analyses of the resulting embryos revealed an obvious abnormal vessel structure at 36 hpf in embryos lacking ATXb regardless of the genotype of *atxa* (Figure 1B), i.e., the same vascular phenotype was observed in ATXa/ATXb DKO (*atxa*^−/−^/*atxb*^−/−^ *ro1* line) embryos (Figure 1B) and ATXb KO embryos (both *ro1* (Figure 1B) and *ro2* lines (Figure S5)). CVP at this stage had column structures, which are endothelial cell-free stromal areas in the CVP (Figure 1A). Wild-type and *atxb*^+/−^ embryos (*atxa*^+/+^/*atxb*^+/+^, *atxa*^−/−^/*atxb*^+/+^, *atxa*^+/+^/*atxb*^+/−^ and *atxa*^−/−^/*atxb*^+/−^) had abundant column structures (Figure 1B, arrowheads). On the other hand, ATXb KO embryos (*atxa*^+/+^/*atxb*^−/−^ and *atxa*^−/−^/*atxb*^−/−^) had fewer and smaller column structures (Figure 1B). Of note, the column structures were seldomly observed in the anterior part in the ATXb KO embryos (Figure 1B). Because we did not observe significant changes in the CVP phenotype among the ATXb KO (*ro1* line), ATXb KO (*ro2* line) and ATXa/ATXb DKO (*ro1* line) embryos (Figure 1B), the subsequent analysis was essentially focused on ATXb KO (*ro1* line) fishes. Quantitative analysis of the column structure in ATXb KO confirmed that the ATXb KO had fewer and smaller column structure (Figures 1C–1E). The total vessel areas as judged by the projection views from the lateral sides (Figure 1B) were comparable between *atxb*^−/−^ and *atxb*^+/+^ embryos (Figure 1F).

Video S1. Time-lapse microscopic image of wild-type embryo (*atxb*^+/+^) taken around 36 hpf, related to Figure 1

Video S2. Time-lapse microscopic image of ATXb KO embryo (*atxb*^−/−^) taken around 36 hpf, related to Figure 1

Cross-sectional views of the CA and CVP showed that they were finely subdivided and separated from each other in wild-type and ATXa KO embryos (Figure 1G, first and second lines, lower panel). By contrast, such vessel subdivision and separation were not obvious in ATXb KO embryos (Figure 1G, third line, lower panel). For example, although CA was lumenized and independent from CVP in the anterior parts (Figure 1G, lower panel, somites a–d), it was continuous with CVP on the posterior parts (Figure 1G, lower panel, somites e–j). The abnormal CA and CVP structures were also confirmed by the observation that a number of blood cells return to the vein without reaching the tail (Video S2), showing that ATXb KO embryos had an abnormal aorta and vein that were not separated as independent vessels. A similar but more severe lack of vessel subdivision was also observed in the anterior parts of CVP (Figure 1G, bottom left, lower panel). In somite a, for example, dCVP and vCVP fused to form a large sac-like vessel, although CA was separated from CVP. In somites b–e, dCVPs fused to form a large sac-like vessel, although they were separated from vCVP (Figure 1G, bottom line, lower panel). We counted the number of subdivided vessels and confirmed that the ATXb KO embryos had a less subdivided CVP, especially in the anterior parts (Figures 1H–1J).

We also examined the CVP formation when ATX was inactivated pharmacologically. The ATX inhibitor ONO-8430506, which was recently developed against mammalian ATX (Iwaki et al., 2020), was found to inhibit both ATXa and ATXb (Kise et al., 2019). When fertilized eggs were treated with the ATX inhibitor from 25 hpf, a CVP phenotype similar to that in ATXb KO embryos was observed at 36 hpf (Figures 1K and S6), confirming that the CVP phenotype in ATX mutants is not an off-target effect.

**Figure 2:**
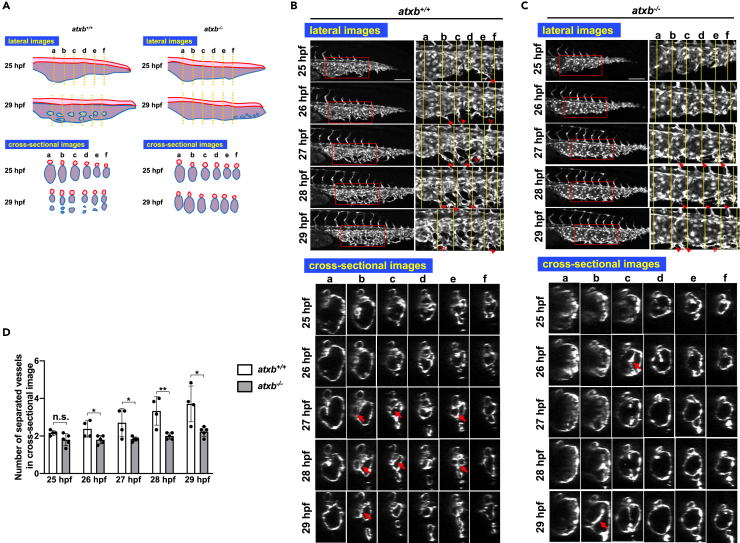
Abnormal vessel segmentation in ATXb KO embryos (A) Schematic diagrams explaining the phenotype of ATXb KO embryos. DA and CVP are drawn in red and blue, respectively. The diagrams for cross-sectional images at the yellow dot line (lower panel) are shown, indicating that ATXb KO embryos have large and sac-like CVP. (B and C) Sequential time-lapse images of wild-type (*atxb*^*+/+*^) (B) and *atxb*^*−/−*^ (C) embryos at the indicated time points. Both projection views from the lateral side (upper panel) and cross-sectional images (lower panel) are shown. Enlarged images of the area surrounded by squares in lateral images are positioned in the right side (upper panel). In *atxb*^*+/+*^ embryos, endothelial cells (ECs) sprout ventrally from the CV (dCVP primordia) and anastomose each other (upper panel). Arrowheads and hollow arrowheads indicate sprouting and anastomosing (rejoining) EC cells, respectively. The sectional images at the six somites (a to f) (lower panel) show that the subdivision of dCVP proceeds in time dependent manner. In this process, ECs sprout into the CV lumen and form a cross-linked (bridging) structure (shown by arrows). Then, CV constriction and subdivision proceed in parallel. In an *atxb*^*−/−*^ embryo (C), we observed extremely little vessel subdivision, which results in remaining of large lumens. Note that both EC sprouting and the sign of forming the bridging structure are still observed, even less frequently. Scale bars, 100 μm. (D) Numbers of separated vessels surrounded by endothelial cells in a cross section (somite a–f in Figures 2B and 2C) at the indicated timepoints. Four wild-type (*atxb*^+/+^) and five ATXb homozygous (*atxb*^−/−^) embryos were evaluated. All data were expressed as means and SD. p value was calculated by the student’s t test (∗p < 0.05; ∗∗p < 0.01; n.s., no significance). See also Video S3. Time-lapse fluorescent microscopic image of wild-type Tg(fli1:EGFP) embryo in a lateral view, related to Figure 2, Video S4. Time-lapse fluorescent microscopic image of wild-type Tg(fli1:EGFP) embryo in a cross-sectional view, related to Figure 2, Video S5. Time-lapse fluorescent microscopic image of ATXb KO (atxb−/−) Tg(fli1:EGFP) embryo in a lateral view, related to Figure 2.

We further observed vessel formation in ATXb KO embryos from 36 hpf back in time. In wild-type embryos, a primitive CV has formed from the CA by 25 hpf (Figures 2A and 2B). Then, from the primitive CV fine dCVP and vCVP were formed by 29 hpf, especially in somite b-f (Figure 2B; Video S3 (lateral view) and S4 (cross-sectional view)). In this process, ventral endothelial cells in the primitive CV had multiple protrusions (sprouting), which anastomosed to form a finely subdivided CVP and multiple column structures (Video S3), or a primitive CV divided into multiple compartments by intussusception (Video S4). By contrast, in ATXb KO embryos, such transformation of vessels was seldom observed (Figures 2A and 2C; Videos S5 (lateral view) and S6 (cross-sectional view)), and as a result, subdivision of vessels (CVP formation) was mostly absent in ATXb KO embryos (Figures 2A, 2C, and 2D). It should be noted here that the extension of endothelial cells (sprouting) and CVP formation were less affected in the posterior part of ATXb KO embryos (Figure 2C and Video S5 (lateral view)).

Video S3. Time-lapse fluorescent microscopic image of wild-type *Tg(fli1:EGFP)* embryo in a lateral view, related to Figure 2 From 25 hpf, time-lapse images of wild-type embryos (*atxb*^+/+^) were taken for every 20 min for 4 h

Video S4. Time-lapse fluorescent microscopic image of wild-type *Tg(fli1:EGFP)* embryo in a cross-sectional view, related to Figure 2 From 26 hpf, time-lapse fluorescent images of wild-type embryos (*atxb*^+/+^) were taken for every 20 min for 4 h

Video S5. Time-lapse fluorescent microscopic image of ATXb KO (*atxb*^−/−^) *Tg(fli1:EGFP)* embryo in a lateral view, related to Figure 2 From 25 hpf, time-lapse images of ATXb KO embryos (*atxb^-^*^/-^) were taken for every 20 min for 4 h

Video S6. Time-lapse fluorescent microscopic image of ATXb KO (*atxb*^−/−^) *Tg(fli1:EGFP)* embryo in a cross-sectional view, related to Figure 2 From 26 hpf, time-lapse fluorescent images of ATXb KO embryos (*atxb^-^*^/-^) were taken for every 20 min for 4 h

### Malformation of CVP in *lpar6a*/*lpar6b* and *gna13a*/*gna13b* double mutant embryos and in embryos treated with inhibitors for Gα_13_ signaling

**Figure 3:**
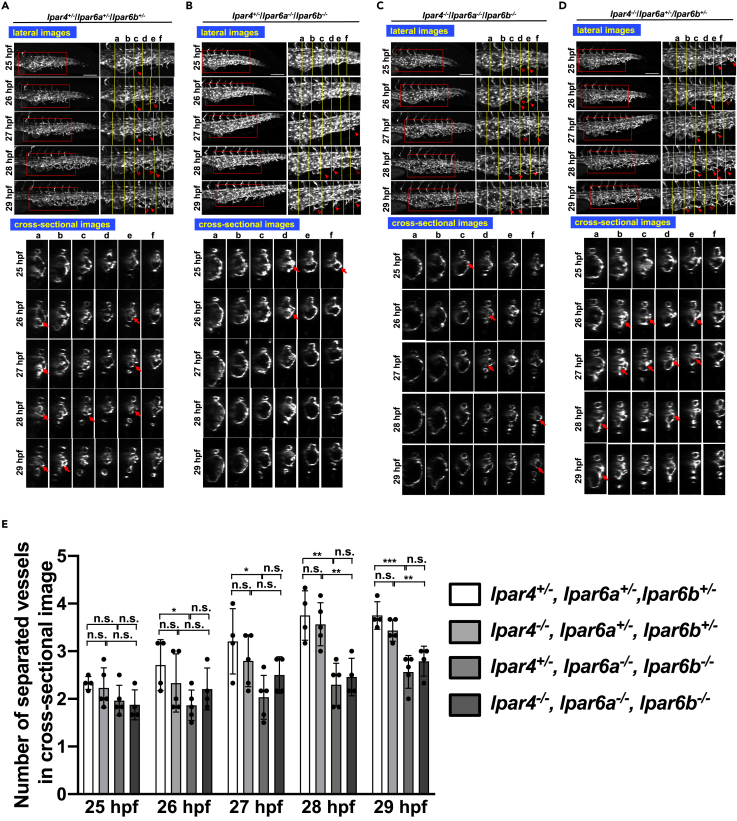
Similar abnormal CVP structure in *lpar6a*/*lpar6b* double mutant embryos (A–D) Sequential time-lapse images of control (*lpar4*^*+/−*^*/lpar6a*^+/−^*/lpar6b*^+/−^) (A) and *lpar4*^*+/−*^*/lpar6a*^*−/−*^*/lpar6b*^−/−^ (B), *lpar4*^*−/−*^*/lpar6a*^*−/−*^*/lpar6b*^−/−^ (C), and *lpar4*^*−/−*^*/lpar6a*^*+/*−^*/lpar6b*^+/−^ (D) embryos at the indicated time points. Both projection views from the lateral side (upper panel) and sectional images (lower panel) are shown. Enlarged images of the area surrounded by squares are positioned in the right side (upper panel). In all embryos, endothelial cell (EC) sprouts and their anastomosis were observed although less frequent in *lpar4*^*+/−*^*/lpar6a*^*−/−*^*/lpar6b*^−/−^ (B) and *lpar4*^*−/−*^*/lpar6a*^*−/−*^*/lpar6b*^−/−^ (C) embryos. Arrowheads and hollow arrowheads indicate sprouts and anastomosed (re-joined) sprouts, respectively. Cross-linked structure formed in lumen is pointed with arrows. Vessel subdivision was significantly attenuated in *lpar4*^*+/−*^*/lpar6a*^*−/−*^*/lpar6b*^−/−^ (B) and *lpar4*^*−/−*^*/lpar6a*^*−/−*^*/lpar6b*^−/−^ embryos (C), which resulted in the remaining large lumens, as was observed for *atxb*^−/−^ mutants. Note that both EC sprouting and the sign of forming the bridging structure are still observed, even less frequently. Scale bars, 100 μm. (E) Numbers of separated vessels surrounded by endothelial cells in a cross section (somite a-f in Figures 3A–3D) at the indicated timepoints. Four or five embryos with genotypes shown were evaluated (*lpar4*^*+/−*^*lpar6a*^+/−^*lpar6b*^+/−^ n = 4, *lpar4*^*−/−*^*lpar6a*^+/−^*lpar6b*^+/−^ n = 5, *lpar4*^*+/−*^*lpar6a*^*−/−*^*lpar6b*^*−/−*^ n = 5, *lpar4*^*−/−*^*lpar6a*^*−/−*^*lpar6b*^*−/−*^ n = 4). All data were expressed as means and SD. p value was calculated by the student’s t test (∗p < 0.05; ∗∗p < 0.01; ∗∗∗p < 0.001; n.s., no significance). See also Figure S7.

**Figure 4:**
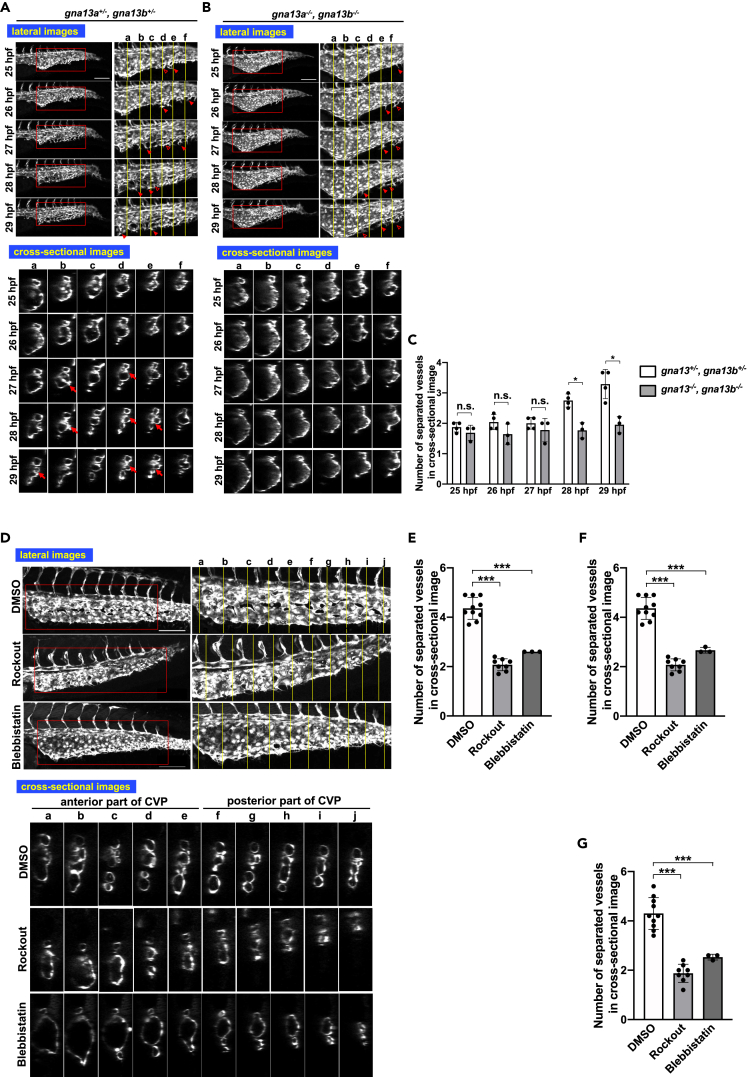
Similar abnormal CVP structure in *gna13a*/*gna13b* double mutant embryos and embryos treated with inhibitors of actin stress fiber formation (A, B, and D) All images are projection views of confocal z stack images and cross-sectional images of CVP. Scale bars, 100 μm. For (D), inhibitors were added at 24 hpf and images were taken at 36 hpf. (A and B) Images of control (*gna13a*^+/−^/*gna13b*^+/−^) and *gna13a*/*gna13b* double KO (*gna13a*^−/−^/*gna13b*^−/−^) embryos were taken from 25 hpf to 29 hpf. Arrowheads and hollow arrowheads indicate sprouts and anastomosed (re-joined) sprouts, respectively. Cross-linked structure formed in lumen is pointed with arrows. (C) Numbers of separated vessels surrounded by endothelial cells in a cross section (somite a–f in A and B) at the indicated timepoints. Four *gna13a*^+/−^/*gna13b*^+/−^ and three *gna13a*^−/−^/*gna13b*^−/−^ embryos were evaluated. All data were expressed as means and SD. p value was calculated by the student’s t test (∗p < 0.05; ∗∗p < 0.01; n.s., no significance). (D) Embryo treated with DMSO only (negative control), ROCK inhibitor (Rockout), or inhibitor of stress fiber formation (Blebbistatin). Note that the vessel subdivision is rarely observed in the anterior somites in *gna13a*^−/−^/*gna13b*^−/−^ embryos and in embryos treated with Rockout and Blebbistatin. (E–G) Numbers of separated vessels surrounded by endothelial cells in a cross section (somite a–f in D) from three to ten embryos (DMSO n = 10, Rockout n = 3, Blebbistatin n = 8). The anterior somites a–e (E), the posterior somites f–j (F) and all somites (a–j) (G) were evaluated. All data were expressed as means and SD. p value was calculated by the student’s t test (∗∗∗p < 0.001). See also Figure S8.

Previous *in vitro* analyses using endothelial cells have shown that LPA induces actin stress fiber formation via LPA_6_ receptor and downstream RhoA and Rho kinase (Yukiura et al., 2015). Furthermore, LPA_4_/LPA_6_ receptor DKO mice (Yasuda et al., 2019), Gα_13_ KO mice (Ruppel et al., 2005), and Rho kinase KO mice (Kamijo et al., 2011) commonly exhibited an embryonic lethal phenotype similar to that of ATX KO mice (Tanaka et al., 2006; van Meeteren et al., 2006), because of defects of embryonic vessel formation. Therefore, we hypothesized that these molecules might also function downstream of ATX in zebrafish, contributing to CVP formation. To test this possibility, we generated LPA_4_, LPA_6_a, and LPA_6_b KO fishes along with their multiple KO fishes as well as Gα_13_a and Gα_13_b KO, and Gα_13_a/Gα_13_b DKO fishes using TALEN and CRISPR Cas9 systems (Figures S7 and S8). Among zebrafish embryos with various genotypes obtained by intercrossing LPA_4_/LPA_6_a/LPA_6_b triple mutants (*lpar4*^−/−^/*lpar6a*^−/−^/*lpar6b*^−/−^ × *lpar4*^+/−^/*lpar6a*^+/−^/*lpar6b*^+/−^) embryos showed the CVP phenotype similar to that observed in the ATX mutants regardless of the LPA_4_ genotype (Figures 3A–3C). *lpar4*^−/−^/*lpar6a*^+/−^/*lpar6b*^+/−^ embryos did not show the phenotype (Figure 3D). We also determined the number of separated vessels in cross-sectional views (Figure 3E), showing that LPA_6_ (both LPA_6_a and LPA_6_b) but not LPA_4_ were needed for proper CVP formation. Essentially the same CVP phenotype was observed in Gα_13_a/Gα_13_b DKO fishes (*gna13a*^−/−^/*gna13b*^−/−^) (Figures 4A–4C).

We further examined whether inhibition of downstream signaling of Gα_13_ led to CVP malformation. Treatment of embryos with inhibitors for Rho kinase (Rockout) and a myosin II (Blebbistatin) after 24 hpf caused CVP abnormalities similar to those observed in ATXb KO, LPA_6_a/LPA_6_b DKO, and Gα_13_a/Gα_13_b DKO embryos (Figures 4D–4G). These analyses strongly suggested that the ATX-LPA_6_-Gα_13_ axis regulated CVP formation by activating downstream RhoA and Rho kinase and the following actin polymerization.

To confirm that the actin cytoskeleton was really affected by ATX inactivation, we used a transgenic (Tg) zebrafish line (*Tg(fli1:lifeact-mCherry)*) expressing mCherry-tagged lifeact, a small actin-binding peptide, under the control of the endothelial cell-specific *fli1* promoter, which enabled the real-time observations of actin dynamics. When the ATX inhibitor ONO-8430506 was administered at 25 hpf, many punctate signals were detected at 36 hpf when abnormal CVP structure was observed, which were never observed in control untreated embryos (Figure S9). This analysis revealed that an ATX-LPA axis actually regulates actin cytoskeleton formation in CVP.

### ATX contributes to the maintenance of CVP

**Figure 5:**
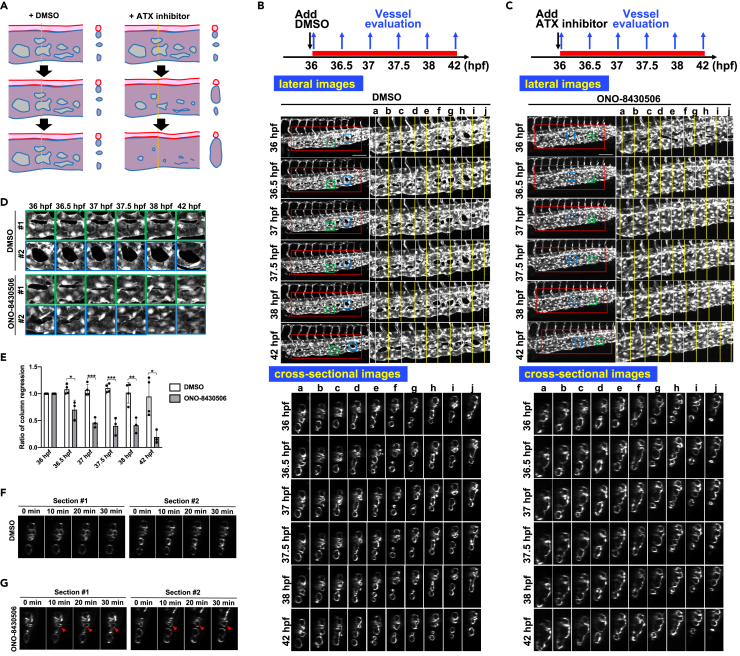
ATX has a role in maintaining CVP structure (A) Schematic diagram explaining the effect of ATX inhibition on CVP structure. DA and CVP are drawn in red and blue, respectively. The diagram for the cross-sectional image at the yellow dot line (right panel) is shown. (B and C) Projection views of confocal z stack images and cross-sectional images of CVP during 36–42 hpf. Wild-type embryos were treated with ATX inhibitor, ONO-8430506, from 36 to 42 hpf and confocal CVP images were taken at indicated time points. Schematic diagrams of the protocol are also shown in the upper side. Images from DMSO (B) or ONO-8430506 (C)-treated embryos are shown. Time-lapse images were shown every 30 min. Scale bars, 100 μm. (D and E) ATX inhibitor treatment induces rapid shrinkage of column structure. (D) Enlarged images of the column structure in the area surrounded by squares in Figures 5B and 5C, showing that the column structure rapidly regresses in a time-dependent manner after ATX inhibitor treatment. (E) Quantitative analysis of the column shrinkage after ATX inhibitor treatment (DMSO n = 4, ONO-8430506 n = 3). Time-dependent changes in the column area. The column area at indicated time points was divided by the column area at 36 hpf, and the resulting relative column area was shown. All data were expressed as means and SD. p value was calculated by the student’s t test (∗p < 0.05; ∗∗p < 0.01; ∗∗∗p < 0.001). (F and G) Rapid CVP expansion in the early phase after ATX inhibitor treatment. At 36 hpf, wild-type embryos were treated with ATX inhibitor, and time-dependent changes of CVP structure in cross-sectional images were taken every 10 min. Arrowheads show the expanded CVP vessels. See also Figure S10 and Video S7. Time-lapse fluorescent microscopic image of wild-type Tg(fli1:EGFP) embryo treated with DMSO in a lateral view, related to Figure 5, Video S8. Time-lapse fluorescent microscopic image of wild-type Tg(fli1:EGFP) embryo treated with DMSO in a cross-sectional view, related to Figure 5, Video S9. Time-lapse fluorescent microscopic image of wild-type Tg(fli1:EGFP) embryo treated with ONO-8430506 (dissolved in DMSO) in a lateral view, related to Figure 5, Video S10. Time-lapse fluorescent microscopic image of wild-type Tg(fli1:EGFP) embryo treated with ONO-8430506 in a cross-sectional view, related to Figure 5, Video S11. Effect of ONO-8430506 treatment on blood flow, related to Figure 5, Video S12. Effect of ONO-8430506 treatment on blood flow, related to Figure 5.

Administration of ATX inhibitors enabled us to verify the function of ATX at any given time. Interestingly, unlike DMSO-treated control embryos (Figures 5A and 5B; Videos S7 and S8), administering ATX inhibitor at 36 hpf, when the CVP had pre-formed, caused many fine vessels to fuse to form larger vessels (Figures 5A and 5C; Videos S9 and S10). The fusion of fine vessels was evident in the anterior parts, and was consistent with the observation that *atxb*^−/−^ embryos had a large and fused CVP in the anterior parts (Figures 1B and 1G). Administration of ONO-8430506 also induced rapid regression of the column structures (Figures 5A, 5D and 5E). The column structures as judged by the areas did not change during the 6 h in DMSO-treated embryos, whereas they rapidly shrank in embryos treated with ONO-8430506. Of note, morphological changes in these CVPs started in as little as 10 min and were clearly observed 20 min after the administration of ATX inhibitor (Figures 5F and 5G), which suggests that the ATX inhibitor’s effect did not involve gene transcription. Although CVP formation was reported to be inhibited by reduced blood flow (Xie et al., 2018), the videos (Videos S11 and S12) strongly suggest that the regression of CVP by ATX inhibition was not due to inhibition of blood flow. We tried to observe the actin cytoskeleton when the column structures in the CVP regressed following ATX inhibition. When *Tg(fli1:lifeact-mCherry)* embryos were treated with the ATX inhibitor ONO-8430506 at 36 hpf, a number of punctate signals were observed 30 min after its administration (Figure S10). The punctate signals were similar to those observed when embryos were treated with the ATX inhibitor at 25 hpf (Figure S9). Together, these data suggested that ATX had a role in maintaining the fine vessel structures, in addition to a role in CVP formation, possibly via the actin cytoskeleton.

Video S7. Time-lapse fluorescent microscopic image of wild-type *Tg(fli1:EGFP)* embryo treated with DMSO in a lateral view, related to Figure 5 At 36 hpf, wild-type embryos were treated with DMSO and time-lapse images were taken for every 30 min for 6 h

Video S8. Time-lapse fluorescent microscopic image of wild-type *Tg(fli1:EGFP)* embryo treated with DMSO in a cross-sectional view, related to Figure 5 At 36 hpf, wild-type embryos were treated with DMSO and time-lapse images were taken for every 30 min for 6 h

Video S9. Time-lapse fluorescent microscopic image of wild-type *Tg(fli1:EGFP)* embryo treated with ONO-8430506 (dissolved in DMSO) in a lateral view, related to Figure 5 At 36 hpf, wild-type embryos were treated with ONO-8430506 and time-lapse images were taken for every 30 min for 6 h

Video S10. Time-lapse fluorescent microscopic image of wild-type *Tg(fli1:EGFP)* embryo treated with ONO-8430506 in a cross-sectional view, related to Figure 5 At 36 hpf, wild-type embryos were treated with ONO-8430506 and time-lapse images were taken for every 30 min for 6 h

Video S11. Effect of ONO-8430506 treatment on blood flow, related to Figure 5 The movie shows blood flow before treating the embryo with ONO-8430506

Video S12. Effect of ONO-8430506 treatment on blood flow, related to Figure 5 The movie shows the blood flow 20 min after treating the embryo with ONO-8430506

### LPA_6_-dependent constriction of the caudal vein plexus (CVP) by an LPA stable analog

We then asked how the ATX-LPA_6_ axis contributes to the formation and maintenance of subdivided vessels. To answer this question, we injected OMPT (1-oleoyl-2--methyl-*sn*-glycero-3-phosphothioate), a stable and potent LPA_6_ agonist (Yanagida et al., 2009; Jiang et al., 2013), into the fish embryos and observed the vessels. To assess whether OMPT reached each part of the vessel, we mixed a dye (Evans blue) with the OMPT solution and injected the mixture in the vicinity of the heart. At 36 hpf when CVP was pre-formed, as soon as OMPT reached CVP, the CVP rapidly shrank (Figure S11B; Video S14), whereas the shrinkage was less in the vehicle control (Figure S11A; Video S13).

**Figure 6:**
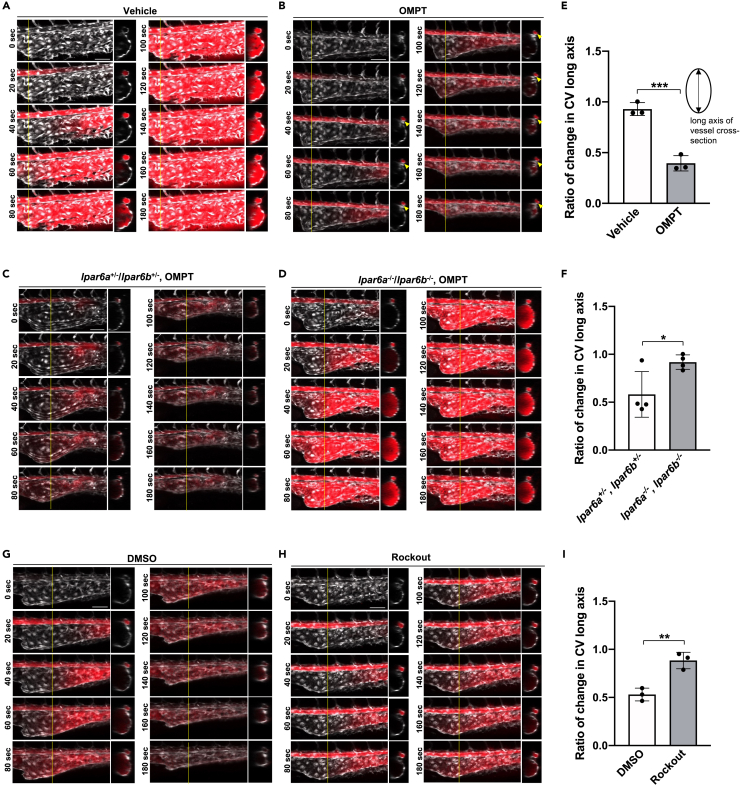
LPA_6_-dependent constriction of caudal vein plexus (CVP) by an LPA stable analog (A and B) Constriction of CVP induced by OMPT. At 25 hpf, wild-type embryos were treated with ATX inhibitor, ONO-8430506 (100 μM), for eleven hours. At 36 hpf, the embryos were injected with OMPT in the vicinity of the heart. Time-lapse images are taken every 20 s after the injection. The circulation of OMPT is evaluated by the fluorescence of Evans Blue, which is mixed with OMPT. The time-lapse images show that Evans Blue and thus OMPT pass through CA and then reach CVP gradually after they enter the circulation. (B) OMPT rapidly induces shrinkage of CVP as soon as it reaches CVP (B), which is never observed in vehicle control (DMSO, A). OMPT also induces the constriction of CA (arrowheads) (B). Scale bars, 50 μm. (C and D) Constriction of CVP induced by OMPT in *lpar6a*^−/−^/*lpar6b*^−/−^ embryos. Treatment with ATX inhibitor, OMPT injection, and analyses were performed as in A and (B). OMPT rapidly induces shrinkage of CVP in *lpar6a*^+/−^/*lpar6b*^+/−^ embryos (C), which was significantly weakened in *lpar6a*^−/−^/*lpar6b*^−/−^ embryos (D). Scale bars, 50 μm. (E and F) Quantitative evaluation of CVP constriction. The length of the CVP long axis was determined using Zen software from the sectional images of confocal z stack images, and the rate of change in CVP long axis was calculated by dividing the length at time 300 s by that at 0 s (E; OMPT vs. vehicle control (DMSO) and F; *lpar6a*^+/−^/*lpar6b*^+/−^ vs. *lpar6a*^−/−^/*lpar6b*^−/−^). Data were shown as means ± SD of three-vehicle control and three OMPT-injected embryos for A and B, respectively, and four *lpar6a*^+/−^/*lpar6b*^+/−^ and four *lpar6a*^−/−^/*lpar6b*^−/−^ embryos, respectively. p value was calculated by the student’s t test (∗p < 0.05; ∗∗∗p < 0.001). (G–I) Effect of Rho-kinase inhibitor on OMPT-induced vasoconstriction. Thirty-four hpf embryos were pre-treated with Rockout (100 μM), and OMPT-induced vasoconstriction was evaluated at 36 hpf as in A and B (G, DMSO control and H, Rockout). Scale bars, 50 μm. (I) Quantitative evaluation of CVP constriction was performed as in (E) and (F). Three embryos treated with DMSO and three embryos treated with Rockout were evaluated. All data were expressed as means and SD. p value was calculated by the student’s t test (∗∗p < 0.01). See also Figures S11, S12 and Video S13. LPA6 agonist-induced rapid vasoconstriction (vehicle control), related to Figure 6, Video S14. LPA6 agonist-induced rapid vasoconstriction (OMPT), related to Figure 6, Video S15. LPA6 agonist-induced vasoconstriction in the embryos pre-treated with ONO-8430506 (vehicle control), related to Figure 6, Video S16. LPA6 agonist-induced vasoconstriction in the embryos pre-treated with ONO-8430506 (OMPT), related to Figure 6, Video S17. LPA6 agonist-induced vasoconstriction is weaken in LPA6a/LPA6b DKO embryos (control, lpar6a+/−/lpar6b+/−), related to Figure 6, Video S18. LPA6 agonist-induced vasoconstriction is weaken in LPA6a/LPA6b DKO embryos (LPA6a/LPA6b DKO, lpar6a−/−/lpar6b−/−), related to Figure 6, Video S19. LPA6 agonist-induced vasoconstriction is weakened in the embryos pre-treated with ROCK inhibitor (control, DMSO), related to Figure 6, Video S20. LPA6 agonist-induced vasoconstriction is weakened in the embryos pre-treated with ROCK inhibitor (ROCK inhibitor, Rockout), related to Figure 6.

Video S13. LPA_6_ agonist-induced rapid vasoconstriction (vehicle control), related to Figure 6 At 36 hpf when CVP was pre-formed, 1nL of 0.1% BSA/PBS containing 0.2% Evans blue was injected into wild-type embryos as a vehicle control and time-lapse images were taken for 5 min every 20 s

Video S14. LPA_6_ agonist-induced rapid vasoconstriction (OMPT), related to Figure 6 At 36 hpf when CVP was pre-formed, 1 nL of 0.1% BSA/PBS containing 0.2% Evans blue and 1 mM OMPT was injected into wild-type embryos and time-lapse images were taken for 5 min every 20 s

We then tried to evaluate OMPT-induced vasoconstriction in LPA_6_a/LPA_6_b DKO embryos. However, because the CVP structures were quite different between LPA_6_a/LPA_6_b DKO and control embryos at 29 hpf (Figure 3), we could not evaluate the effect of OMPT on vasoconstriction at this time point. At 25 hpf, although the vessel structures in both wild-type and *lpar6a*^−/−^/*lpar6b*^−/−^ embryos were similar, the heart beats weakly, and the injected OMPT did not circulate well in the bodies (data not shown). When treated with the ATX inhibitors at 25 hpf, embryos had similar enlarged CVP structures at 36 hpf, regardless of the genotype (Figures 6A–6D, time 0 s). Therefore, we treated the embryos with ATX inhibitor at 25 hpf and injected OMPT at 36 hpf. In wild-type embryos, injection of OMPT but not PBS (vehicle control) at 36 hpf rapidly induced vasoconstriction of the CVP (Figures 6A and 6B; Videos S15 and S16). The OMPT-induced rapid CVP constriction was observed in *lpar6a*^+/−^/*lpar6b*^+/−^ but not in *lpar6a*^−/−^/*lpar6b*^−/−^ embryos (Figures 6C and 6D; Videos S17 and S18). Quantitative analysis of the long axis of the vessel cross-section confirmed that OMPT-induced vasoconstriction is significantly suppressed in *lpar6a*^−/−^/*lpar6b*^−/−^ embryos (Figures 6E and 6F). We also confirmed that pretreatment of the embryos with Rockout significantly inhibited the OMPT-induced vasocontraction, indicating that the vasocontraction is ROCK-dependent (Figures 6G–6I, and Videos S19 and S20). In addition, treatment of *Tg(fli1:lifeact-mCherry)* with OMPT caused the punctate signals to disappear (Figure S12). These punctate signals were induced by treatment of ATX inhibitors (Figures S10 and S12). These analyses revealed that OMPT-induced activation of LPA_6_ and the downstream Rho kinase led to CVP constriction via modification of the actin cytoskeleton.

Video S15. LPA_6_ agonist-induced vasoconstriction in the embryos pre-treated with ONO-8430506 (vehicle control), related to Figure 6 At 25 hpf embryos were treated with ONO-8430506 and at 36 hpf they were injected with 0.1% BSA/PBS containing 0.2% Evans blue (vehicle control). Time-lapse images were taken for 5 min every 20 s

Video S16. LPA_6_ agonist-induced vasoconstriction in the embryos pre-treated with ONO-8430506 (OMPT), related to Figure 6 At 25 hpf embryos were treated with ONO-8430506 and at 36 hpf they were injected with 0.1% BSA/PBS containing 0.2% Evans blue and 1 mM OMPT (OMPT). Time-lapse images were taken for 5 min every 20 s

Video S17. LPA_6_ agonist-induced vasoconstriction is weaken in LPA_6_a/LPA_6_b DKO embryos (control, *lpar6a*^+/−^*/lpar6b*^+/−^), related to Figure 6 At 25 hpf *lpar6a*^+/−^/*lpar6b*^+/−^ embryos were treated with ONO-8430506 and at 36 hpf they were injected with 0.1% BSA/PBS containing 0.2% Evans blue and 1 mM OMPT. Time-lapse images were taken for 5 min every 10 s

Video S18. LPA_6_ agonist-induced vasoconstriction is weaken in LPA6a/LPA6b DKO embryos (LPA_6_a/LPA_6_b DKO, *lpar6a*^−/−^*/lpar6b*^−/−^), related to Figure 6 At 25 hpf *lpar6a*^*−/−*^*/lpar6b*^*−/−*^ embryos were treated with ONO-8430506 and at 36 hpf they were injected with 0.1% BSA/PBS containing 0.2% Evans blue and 1 mM OMPT. Time-lapse images were taken for 5 min every 10 s

Video S19. LPA_6_ agonist-induced vasoconstriction is weakened in the embryos pre-treated with ROCK inhibitor (control, DMSO), related to Figure 6 At 25 hpf wild-type embryos were treated with ONO-8430506, at 35.5 hpf were treated with DMSO (control) and at 36 hpf they were injected with 500 μM OMPT. Time-lapse images were taken for 5 min every 20 s

Video S20. LPA_6_ agonist-induced vasoconstriction is weakened in the embryos pre-treated with ROCK inhibitor (ROCK inhibitor, Rockout), related to Figure 6 At 25 hpf wild-type embryos were treated with ONO-8430506, at 35.5 hpf were treated with Rockout (ROCK inhibitor) and at 36 hpf they were injected with 500 μM OMPT. Time-lapse images were taken for 5 min every 20 s

### CVP formation induced by blood flow is dependent on ATX

**Figure 7:**
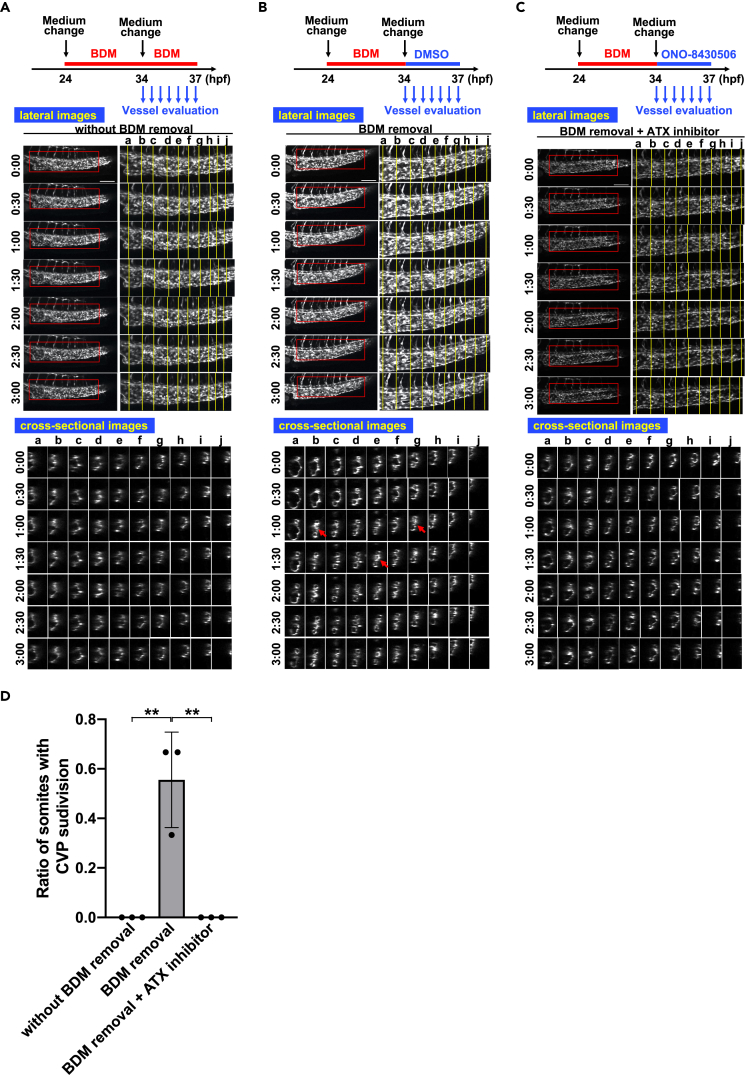
Blood flow-induced CVP formation is dependent on ATX (A) Twenty-four hpf embryos were pre-treated with BDM (12 mM) for ten hours by changing the medium to a medium containing BDM, and at 34 hpf the medium was changed to the same medium containing BDM and the sequential time-lapse images were taken for 3 h every 30 min. The CVP structures did not change significantly for 3 h. Scale bars, 100 μm. (B) Twenty-four hpf embryos were pre-treated with BDM as in (A), and at 34 hpf the medium was changed to a medium without BDM and the sequential time-lapse images were taken for 3 h every 30 min. Note that subdivisions of CVP accompanied by constriction of vessels were observed (arrows). Scale bars, 100 μm. (C) Twenty-four hpf embryos were pre-treated with BDM as in (A), and at 34 hpf the medium was changed to a medium without BDM but containing ONO-8430506, and the sequential time-lapse images were taken for 3 h every 30 min. Note that subdivisions and constriction of CVP were significantly suppressed. Scale bars, 100 μm. (D) Ratio of somites with CVP subdivision was quantified by evaluating ten somites (somite a–j in Figures 7A–7C) from each three embryos. All data were expressed as means and SD. p value was calculated by the student’s t test (∗∗p < 0.01). See also Videos S21 and S22.

Several studies have shown that a similar CVP malformation was induced when the blood flow was suppressed (Goetz et al., 2014; Xie et al., 2018; Karthik et al., 2018). Because inhibition of the ATX-LPA_6_ axis disturbed both CVP formations and blood flow at about the same time (Figure 1; Videos S1, S2, S3, S4, S5, and S6), we attempted to elucidate the relationship between the ATX-LPA_6_ axis and blood flow. Treatment of zebrafish embryos with 2,3-butanedione-2-monoxime (BDM) at 24 hpf, which is known to lower the heart rate, rapidly induced a marked reduction in the heart rate and blood flow, and resulted in the formation of abnormal CVP structures (non-subdivided vessels) at 34 hpf (Karthik et al., 2018; Nagasawa-Masuda and Terai, 2017) (Figure 7A). When BDM was removed at 34 hpf, the blood flow resumed with the recovery of heart rate (Video S21), and the CVP started to subdivide to form fine vessels at 37 hpf (Figure 7B). When BDM was removed but ONO-8430506 was added at 34 hpf, subdivision of the CVP was significantly suppressed (Figures 7C and 7D). It should be noted that the blood flow resumed in the presence of ONO-8430506 (Video S22), which shows that ATX has a role in the formation of the CVP caused by the resumption of blood flow.

Video S21. Resumption of blood flow is not significantly suppressed by ATX inhibition (DMSO control), related to Figure 7 Wild-type embryos were treated with DMSO (around 34 hpf) following removal of BDM. Time-lapse microscopic image was taken 2 h after the removal of BDM

Video S22. Resumption of blood flow is not significantly suppressed by ATX inhibition (ATX inhibitor), related to Figure 7 Wild-type embryos were treated with ONO-8430506 (around 34 hpf) following removal of BDM. Time-lapse microscopic image was taken 2 h after the removal of BDM

## Discussion

### The ATX-LPA axis is essential for blood flow-induced CVP formation

In a developing embryo, after the initial vessel structures have been established, blood flow is particularly important in vascular remodeling, which includes stabilization, diameter adjustment, and regression. In this study, we showed that an ATX-LPA_6_ axis regulates CVP formation in concert with blood flow stimulation. We found that treatment with ATX inhibitors before CVP formation inhibited CVP formation, and treatment with ATX inhibitors after CVP formation prevented the maintenance of CVP structures, including column structures. Furthermore, these abnormalities in the formation and maintenance of the CVP were observed when blood flow was inhibited. Of note, the CVP formation caused by the resumption of blood flow was markedly inhibited by ONO-8430506 (Figure 7C). Thus, ATX, and possibly downstream LPA_6_ signaling, was shown to be essential for the blood flow-driven CVP formation. This result also suggests that either (1) LPA production by ATX is dependent on the blood flow or (2) the blood flow-induced CVP formation is stimulated by the presence of LPA. The former seems unlikely, because LPA production proceeds satisfactorily in a static tube. Regarding the latter explanation, LPA was found to sensitize the shear stress-induced Ca^2+^ response in endothelial cells (Ohata et al., 2011). Thus, it is reasonable to assume that LPA produced by ATX accelerates the blood flow-induced CVP formation through LPA_6_ in zebrafish embryos. This is supported by the finding that CVP formation was accelerated by increasing the blood flow (Karthik et al., 2018). *In vitro* analysis using cultured endothelial cells is needed to clarify how LPA stimulation regulates the morphological changes of endothelial cells induced by blood flow (i.e., induced by shear stress).

### The ATX-LPA_6_ axis contributes to the formation and maintenance of CVP

In this study, we showed that the ATX-LPA_6_ axis contributed to the formation and maintenance of CVP using various zebrafish mutants (ATX, LPA_6_, and Gα_13_) and inhibitors. In embryos of these mutants or those treated with inhibitors, the process of CVP formation was abnormal; especially, the process with subdivision of blood vessels was impaired. On the other hand, treatment of pre-formed CVP with inhibitors resulted in impairment of CVP structure and reversion to a more primitive CVP, i.e., a large and sac-like CVP. Interestingly similar vessel abnormalities were observed in ATX KO (Tanaka et al., 2006; van Meeteren et al., 2006) and LPA_4_/LPA_6_ DKO (Yasuda et al., 2019) mice, which had numerous large vessels in the yolk sac and brain. Thus, it is likely that the ATX-LPA receptor axis has a conserved role in a wide range of animal species. ATX expression is especially high in typical plexuses such as vessels in the choroid plexus in the brain (Savaskan et al., 2007) and high endothelial venules in the lymph node (Nakasaki et al., 2008). Considering that LPA_6_ is expressed widely in endothelial cells (Takara et al., 2017; Yukiura et al., 2015), ATX-LPA_6_ may contribute to the formation and maintenance of such vascular plexuses.

In the present zebrafish model, we could not determine the role of LPA_4_, because loss of LPA_4_ did not affect the CVP phenotype in embryos (Figure 3). In both mammals and zebrafish, the six LPA receptors (LPA_1_ to LPA_6_) and five sphingosine 1-phosphate (S1P) receptors (S1P_1_ to S1P_5_) are conserved, whereas some of them are duplicated in zebrafish. Thus, although these lysophospholipid receptors are genetically conserved, they may have slightly different roles in mammals and fish, as was recently reported for S1P receptors (Hisano et al., 2015).

### The ATX-LPA_6_ axis acts by inducing contractile force in endothelial cells

**Figure 8:**
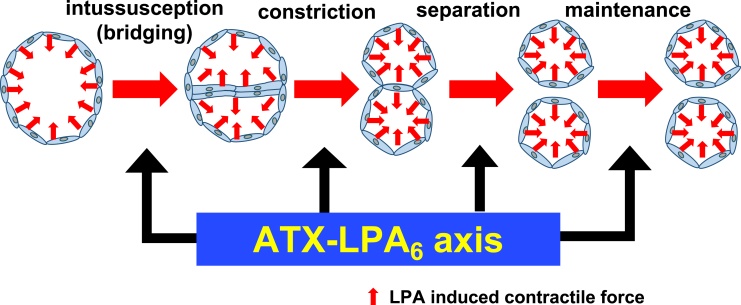
A proposed model explaining the role of ATX-LPA_6_ axis in formation and maintenance of plexus vessels Plexus vessels are formed from pre-existing vessels both by sprouting and intussusceptive angiogenesis and the following constriction and separation of two pre-formed vessels. The present study proposes that an ATX-LPA_6_ axis contributes to vessels' constriction and separation, and also to maintain formed vessels downstream of ATX-LPA_6_-Gα_13_ signaling.

By directly injecting OMPT, an LPA_6_ agonist, into the embryos (*Tg(fli1:EGFP)*), we attempted to verify how the ATX-LPA_6_ axis affects the morphology of endothelial cells. Injection of OMPT induced dramatic changes in the morphology of endothelial cells leading to vasoconstriction in zebrafish embryos and this effect is suppressed in *lpar6a/lpar6b* DKO embryos and embryos treated with ROCK inhibitor (Figure 6). We confirmed that administration of OMPT affected the actin cytoskeleton in CVP (Figure S12). We also previously demonstrated that *i.v.* administration of LPA in mice induced transient hypertension in an LPA_6_/ROCK-dependent manner, possibly by inducing vasoconstriction (Kano et al., 2019). These results suggest that ATX-LPA induces a contractile force on endothelial cells through the LPA_6_-Gα_13_-RhoA-ROCK axis and that vasoconstrictive forces somehow contribute to vessel subdivision and maintenance. Time-lapse observations of subdividing primitive vessels in the CVP revealed that they first underwent shrinking and then separated from each other, forming two independent vessels (Figure 2B; Video S4). As shown in Figure 8, we propose that the contractile force in endothelial cells generated by an ATX-LPA_6_ axis contributes to the formation (intussusception, vessel shrinkage, and separation) and maintenance of the two vessels. We speculate that LPA acts on blood vessels from the lumen side and generates a force in the inward direction because LPA is known to be a blood-derived factor, and when OMPT, a stable derivative of LPA, is in the blood, it rapidly constricts blood vessels.

### ATX-LPA_6_ axis acts by reorganizing the actin cytoskeleton through the Gα_13_-RhoA-ROCK pathway

CVP formation is regulated by several factors that affect actin filament organization (Goetz et al., 2014; Xie et al., 2018; Karthik et al., 2018; Nagasawa-Masuda and Terai, 2017; Choi et al., 2011; Wakayama et al., 2015). In various cell types, including endothelial cells, an LPA-Gα_13_ signal induced actin fiber modification, thereby altering cell morphology, possibly by modulating the adhesive properties of the cells. In addition, several lines of evidence have suggested that actin fiber modification induced by an LPA-Gα_13_ signal stimulates embryonic vessel formation. Indeed, actin polymerization downstream of an LPA_4_/LPA_6_ signal in endothelial cells was recently suggested to stimulate nuclear translocation (activation) of Yap/Taz transcription factors to induce sprouting angiogenesis both *in vivo* and *in vitro* (Yasuda et al., 2019). Thus, it was assumed that the ATX-LPA_6_ axis regulates CVP formation through the Gα_13_-RhoA-ROCK pathway. In this study, we showed that knockout or inhibition of each component of an ATX-LPA signal involved in actin stress fiber formation, *i.e.,* ATX, LPA_6_, Gα_13_, ROCK, and myosin II, caused a similar CVP phenotype in zebrafish (Figures 1, 2, 3, and 4). These results suggest that each factor functions in the same signaling axis in CVP formation. We also analyzed actin cytoskeleton dynamics in CVP using transgenic (Tg) zebrafish line (*Tg(fli1:lifeact-mCherry)*). Inhibition of ATX somehow altered the state of the actin cytoskeleton in CVP, which was revealed by the detection of punctate actin signals (Figures S9 and S10). Interestingly, these signals were abolished by the injection of OMPT, a stable and potent LPA_6_ agonist, indicating that the appearance of punctate signals is because of the direct effect of ATX inhibition. Similar punctate signals were observed when amotl2 expression was suppressed by MO in zebrafish embryos (Hultin et al., 2014). Amotl2 is a factor involved in actin polymerization. Punctate signals of actin were also detected when human umbilical vein endothelial cells (HUVEC) were treated with the ATX inhibitor ONO-8430506 (data not shown). These results support the idea that the punctate signals appear in association with abnormal actin polymerization.

### The ATX-LPA_6_ axis causes rapid endothelial cell morphological changes

**Figure 9:**
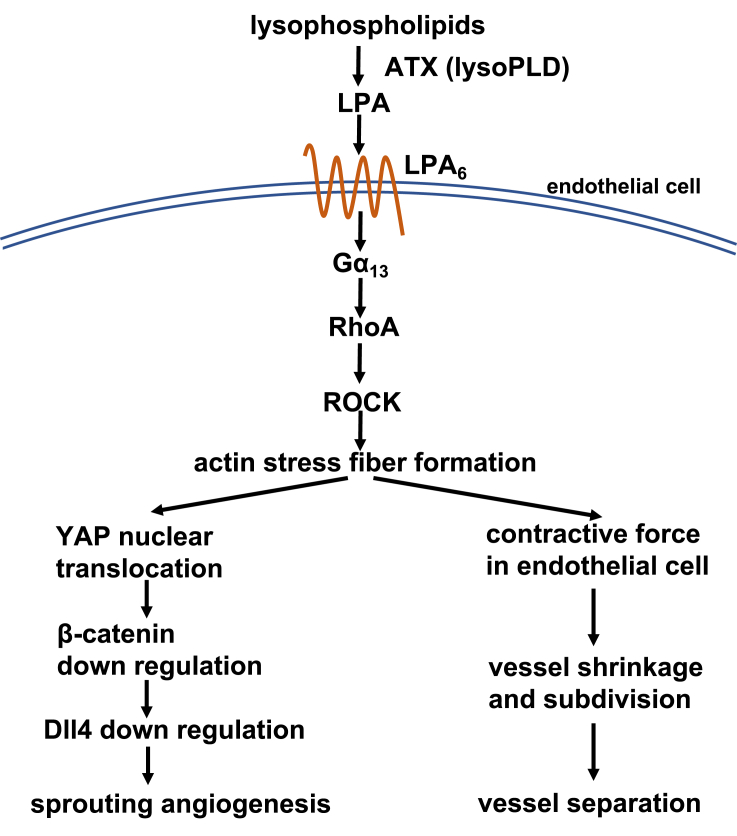
Schematic diagram of the expected function of ATX-LPA_6_-Gα_13_-ROCK axis in CVP formation LPA produced by ATX activates the LPA_6_ receptor on endothelial cells, which induces actin stress fiber formation via Gα_13_, RhoA, and ROCK pathway. Actin stress fiber formation is then stimulated and contributes to (1) activation of Yap transcription factor leading to sprouting angiogenesis via β-catenin and Notch signaling pathways, (2) generation of contractile forces on ECs in developing plexus vessels leading to vessel subdivision and separation.

Addition of ATX inhibitor caused rapid (<20 min) changes in the pre-formed CVP, including loss of column structures and obvious vessel fusion (Figure 5). Moreover, injection of OMPT induced dramatic changes in the morphology of endothelial cells leading to vasoconstriction in zebrafish embryos (Figure 6). Of note, the OMPT-induced vasoconstriction occurred very rapidly, i.e., within 20 s (Figure 6). These observations strongly support the idea that rapid actin fiber reorganization induced by an ATX-LPA_6_ axis contributed to the formation and maintenance of vessel subdivision. Furthermore, these changes most likely did not involve Yap/Taz transcription factors because control by transcription products generally takes more than 1 h. Thus, in addition to the regulation of the Yap/Taz transcription factor proposed by Yasuda et al. (2019), actin stress fiber formation induced by ATX-LPA_6_ signaling induces a morphological change in endothelial cells, which contributes to the formation and maintenance of the CVP in zebrafish (Figure 9).

### Zebrafish ATX mutants are not lethal

ATXa/ATXb DKO as well as ATXb KO zebrafish did not show embryonic lethality, unlike ATX KO mice (Figure S2). ATX KO mice showed abnormal formation of blood vessels in the placenta and yolk sac. These vessels are essential for the nutrient supply from the mother to the embryos, and are the main reason why ATX KO is embryonically lethal. On the other hand, in the early stages of zebrafish development, nutrients are supplied to the whole body by free diffusion from the yolk, and early vascular dysplasia is not necessarily lethal.

Based on the DNA sequences of the mutant zebrafish ATX genes, ATX proteins expressed in the ATX mutants are expected to lose their catalytic activity. In fact, we confirmed that plasma lysoPLD activity was almost lost in the KO fish (Figure S3). However, we could not rule out the possibility that truncated ATXb protein with two SMB domains is expressed in the ATXb mutants and suppresses the embryonic lethal phenotype. Precise analysis of mutant ATX proteins will answer the question.

### ATX produces unsaturated acyl-LPA species in zebrafish

We measured the circulating LPA acyl-chain species in the plasma from adult zebrafish; the rank order was 18:2-LPA > 22:6-LPA > 20:5-LPA > 20:4-LPA > 18:1-LPA > 16:0-LPA (Figure S13). Administration of ATX inhibitors revealed that these LPA species were mostly produced by ATX (Figure S13). LPA species with unsaturated fatty acids were previously shown to be potent LPA_6_ agonists (Yanagida et al., 2009). Previous reports have shown that compared to other LPA receptors, LPA_6_ is strongly expressed in some type of endothelial cell such as human umbilical vein endothelial cells (HUVEC) (Yukiura et al., 2015) and primary endothelial cells derived from mouse vessels (Takara et al., 2017). Therefore, we speculate that the LPA species produced in the blood are able to act on LPA_6_ from the lumen side of the blood vessel, as the initial vessel formation is completed, and blood flow is initiated.

In summary, using zebrafish as a model animal to study the mechanisms of blood vessel formation, we revealed an essential role of an ATX-LPA_6_-Gα_13_ axis in the formation and maintenance of caudal vein plexus (CVP) as well as the relationship between blood flow and the axis. Our next goal is to see whether an ATX-LPA_6_-Gα_13_ axis also has a role in blood vessel function in the adult stage.

### Limitations of the study

The present study showed that the ATX-LPA_6_ axis had a critical role in regulating specific kinds of endothelial cells in caudal vein plexus (CVP) in zebrafish. However, it is still unclear whether the same axis has a role in regulating endothelial cells in other parts and other animals. In addition, it is not clear what the ATX-LPA_6_ axis induces cellular events at the cellular level, which requires experiments using endothelial cells in culture.

## STAR★Methods

### Key resources table

**Table:** 

REAGENT or RESOURCE	SOURCE	IDENTIFIER
**Chemicals, peptides, and recombinant proteins**		

Tricaine (Ethyl-3-aminobenzoate methanesulfonate)	Sigma Aldrich	Cat#E10521-10G; CAS: 886-86-2
1-Phenyl-2-thioourea (PTU)	Wako	Cat#166-13702
Agarose, low gelling temperature	Sigma Aldrich	Cat#A9414-5G
ONO-8430506	Ono Pharmaceutical Co., Ltd. Iwaki et al. (2020)	CAS: 1354805-08-5
(R)-alkyl-OMPT	Wako	Cat#L-9618
GenomeCraft Cas9	FASMAC	Cat#GE-005-S
Blebbistatin	Sigma Aldrich	Cat#B0506
Rockout	Calbiochem	CAS: 7272-84-6
2,3-Butanedione monoxime (BDM)	Sigma Aldrich	Cat#B0753-25G; CAS: 57-71-6

**Critical commercial assays**		

mMESSAGE mMACHINE^TM^ T3 kit	Thermo Fisher Scientific	Cat#AM1348
QIAquick PCR Purification kit	Qiagen	Cat#28106
MEGAshortscript^TM^ T7 kit	Thermo Fisher Scientific	Cat#AM1354
BCA^TM^ Protein Assay kit	Thermo Fisher Scientific	Cat#23250

**Experimental models: Organisms/strains**		

Zebrafish: *Tg(fli1:EGFP)*^*y1*^: y1Tg, AB line	ZFIN	ZFIN: ZDB-ALT-011017-8
Zebrafish: *Tg(fli1:lifeact-mCherry)*, AB line	Dr. Yuki Wakayama; Wakayama et al. (2015)	N/A
Zebrafish: *atxa*, AB line	Kise et al. (2019)	N/A
Zebrafish: *atxb*^*ro1*^, AB line	This paper	N/A
Zebrafish: *atxb*^*ro2*^, AB line	This paper	N/A
Zebrafish: *lpar4*^*rk2*^, AB line	This paper	N/A
Zebrafish: *lpar6a*^*ro20*^, AB line	This paper	N/A
Zebrafish: *lpar6b*^*ro23*^, AB line	This paper	N/A
Zebrafish: *gna13a*^*ro8*^, AB line	This paper	N/A
Zebrafish: *gna13b*^*ro10*^, AB line	This paper	N/A

**Oligonucleotides**		

Primer: sgRNA_F: AAAAGCACCGACTCGGTGCCACTTTTTCAAGTTGATAACGG ACTAGCCTTATTTTAACTTGCTATTTCTAGCTCTAAAAC	This paper	N/A
Primer: zATXb_sgRNA_R: TAATACGACTCACTATAGGACTCACGCTCCCAGAATGGTT TTAGAGCTAGAAATAGC	This paper	N/A
Primer: zLPA_6_a_sgRNA_R: TAATACGACTCACTATAGGTGTTTAGCATCGTCTTCAGT TTTAGAGCTAGAAATAGC	This paper	N/A
Primer: zLPA_6_b_sgRNA_R: TAATACGACTCACTATAGGTCAACGCTAATGCACGTGGTT TTAGAGCTAGAAATAGC	This paper	N/A
Primer: zGα_13_a_sgRNA_R: TAATACGACTCACTATAGGTACTCGCTGGATGACACTGTTTT AGAGCTAGAAATAGC	This paper	N/A
Primer: zGα_13_b_sgRNA_R: TAATACGACTCACTATAGGACGGGGATGACTTCGATAGTTTT AGAGCTAGAAATAGC	This paper	N/A
Primer: zATXb_F: TCATGTGGAACTCACGCTCCC	This paper	N/A
Primer: zATXb_R: CAGTGTGTAGAGGTTGGGGAAG	This paper	N/A
Primer: zLPA_6_a_F: GCTCAATGTGAGCAACGTCA	This paper	N/A
Primer: zLPA_6_a_R: AGATGTACATGGCGGCAACA	This paper	N/A
Primers for used for detecting mutation in genes except ATXb and LPA_6_a, see Table S1	This paper	N/A

**Recombinant DNA**		

Plasmid: pRCIscript-GoldyTALEN	Carlson et al. (2012); Addgene	Cat#42654

**Software and algorithms**		

TAL Effector Nucleotide Targeter version 2.0	Cornell University	https://tale-nt.cac.cornell.edu
Optimized CRISPR design	MIT, Zhang Lab	N/A
ZEN 2 (blue edition)	ZEISS	N/A
Prism8	GraphPad	N/A

### Resource availability

#### Lead contact

Further information and requests for resources and reagents should be directed to and will be fulfilled by the lead contact, Junken Aoki (jaoki@mol.f.u-tokyo.ac.jp).

#### Materials availability

Due to an unfortunate accident, we've lost all zebrafish lines. Thus, zebrafish lines generated in this study are not available.

### Experimental model and subject details

#### Zebrafish

Fishes used in this study were AB background transgenic fish, *Tg*(*fli1:EGFP*)^*y1*^ and *Tg(fli1:lifeact-mCherry)*; and AB background mutant lines, *atxa*^*rk1*^, *atxb*^*ro1*^, *atxb*^*ro2*^, *lpar4*^*rk2*^, *lpar6a*^*ro20*^, *lpar6b*^*ro23*^, *gna13a*^*ro8*^ and *gna13b*^*ro10*^. *Tg*(*fli1:EGFP*)^*y1*^ were obtained from the Zebrafish International Resource Center (University of Oregon, Eugene, OR). *Tg(fli1:lifeact-mCherry)* was established in Wakayama et al. (2015). Fishes were maintained at 27–28°C under a controlled 13.5-h light/10.5-h dark cycle. Embryos were obtained from natural spawning and kept in E2 embryo medium (15.0 mM NaCl, 0.5 mM KCl, 1.0 mM MgSO_4_, 0.15 mM KH_2_PO_4_, 0.05 mM Na_2_HPO_4_, 1.0 mM CaCl_2_, 0.7 mM NaHCO_3_) at 27–28°C. For vessel observation, embryos were treated with 0.003% 1-phenyl-2-thiourea (Sigma) from 24 hpf. Adult fishes used for plasma preparation were male and 3 to 12 months old. All animal experiments were performed in accordance with protocols approved by the Institutional Animal Care and Use Committee at Tohoku University and Animal Committees of the University of Tokyo following the Standards Relating to the Care and Management of Experimental Animals in Japan.

### Method details

#### Generation of mutant fish lines

An *Lpar4* mutant was prepared using TALEN-mediated mutagenesis, as described previously. TALEN constructs targeting *lpar4* were designed using online software (TAL Effector Nucleotide Targeter version 2.0, https://tale-nt.cac.cornell.edu). The left and right arms of TALEN targeting sequences were 5′-GGTACGCACTCGTGCACT-3′ and 5′-AGGGTTCTTGCAAGCCTCTCC-3′, respectively. A targeting construct was generated by the Golden Gate assembly method as described previously (Cermak et al., 2011). Module, array, last repeat and backbone plasmids were assembled by BraI digestion and DNA ligation. The destination vector pRCIscript-GoldyTALEN (Carlson et al., 2012) was obtained from Addgene (Watertown, MA). The vector was linearized by SacI digestion, and mRNA was synthesized using mMESSAGE mMACHINE T3 kit (Thermo Fisher Scientific) and purified by lithium chloride precipitation. Forward- and reverse-TALEN mRNAs (400 pg each) were injected together into one-cell-stage embryos.

Zebrafish mutants of *atxb*, *lpar6a*, *lpar6b*, *gna13a*, and *gna13b* were generated using CRISPR as previously described (Ota et al., 2014) with minor modifications. The sgRNAs were designed using Optimized CRISPR design (MIT, Zhang Lab). Template DNAs for the synthesis of sgRNAs were prepared by PCR amplification using oligonucleotide primers (Table S1). The PCR products were purified using a QIAquick PCR Purification kit (Qiagen). sgRNAs were transcribed using MEGAshortscript^TM^ T7 kit (Thermo Fisher Scientific) and purified by phenol-chloroform extraction and EtOH precipitation. sgRNAs were mixed with Cas9 protein (FASMAC) and injected into 1–2 cell stage embryos. Double KO mutants of *lpar6a*/*lpar6b* and *gna13a*/*gna13b* were obtained by injecting two sgRNAs. *atxa*/*atxb* double and *lpar4*/*lpar6a*/*lpar6b* triple mutants were obtained by injecting into *atxa*^-/-^ and *lpar4*^-/-^ embryos, respectively. Each mutation was confirmed by a heteroduplex mobility assay, as previously described (Ota et al., 2013). DNA fragments which include the target site were amplified using primers listed in Table S1 and electrophoresed on 15% polyacrylamide gels. Mutagenesis was evaluated by the appearance of bands derived from heteroduplexes.

#### Reagents

ATX inhibitor, ONO-8430506 (Iwaki et al., 2020) was kindly provided from Ono Pharmaceutical Co., Ltd. ONO-8430506 was dissolved in DMSO and used at ten μM (*in vitro* experiment) or 100 μM (*in vivo* experiment). Blebbistatin was purchased from SIGMA, dissolved in DMSO, and used at 6.25 μM. OMPT ((*R*)-alkyl-OMPT) was dissolved in 0.1%BSA/PBS and was used at 500 or 1000 μM for *in vivo* injection experiments. Rockout was purchased from Calbiochem, dissolved in DMSO, and used at 50 μM.

#### Determination of ATX (lysoPLD) activity

LysoPLD assay was performed as described previously (Kise et al., 2019), except for the incubation time with substrate. For blood collection, adult zebrafish were anesthetized by Tricaine (SIGMA) and cut between the anal fin and the caudal fin with a surgical razor blade. The exuded blood was collected into a tube filled with PBS (100 μl) containing 5U/mL heparin by immersing the fins in the PBS. The blood was centrifuged at 500 × g for 10 minutes, and the supernatant was used as a diluted plasma. Protein concentration was determined by BCA assay (Thermo Fisher Scientific). The diluted plasma (10 μl) was added to lysoPLD assay buffer (total volume 60 μl) containing 14:0 (myristoyl)-LPC (2 mM) and incubated at 37°C. After 48 hours, free choline concentration in the tube was determined by choline colorimetrical assay (Umezu-Goto et al., 2002). The liberated choline was detected by an enzymatic photometric method using choline oxidase, horseradish peroxidase (Toyobo), and TOOS reagent as a hydrogen donor.

#### Measurement of plasma LPA concentration

Intraperitoneal injection of ATX inhibitor into adult zebrafish was performed as described previously (Luukinen et al., 2018). An ATX inhibitor ONO-8430506 in DMSO (100 mM × 7 μL) and vehicle control (DMSO, 7 μL) was injected using 30 G needle. Blood was collected 40 minutes after the administration. For blood collection, adult zebrafish were anesthetized by Tricaine (SIGMA) and cut between the anal fin and the caudal fin with a surgical razor blade. 1 μL of exuded blood was taken from one adult fish and totally 10 μL of blood was collected in 20 μl of PBS containing 5U/mL heparin from 10 adult fishes. For blood collection, we used P10 pipettor with pipette tips, which was coated with heparin in advance. The diluted blood was centrifuged at 1500 × g for 10 minutes at 4°C, and the supernatant was used as diluted plasma. Measurement of LPA in the diluted plasma was performed as recently described (Kano et al., 2021). The methanol extract containing internal standard (100 μM 17:0 LPA) was subjected to LC-MS/MS.

#### Image acquisition, processing, and quantification

Embryos were embedded in a drop of low melting point agarose (1%) containing 0.016% Tricaine and 0.003% 1-phenyl-2-thiourea. Images of embryos were captured with LSM 800 confocal laser-scanning microscope (Carl Zeiss) equipped with Plan-Apochromat 10×/0.45 M27 or Plan-Apochromat 20×/0.8 M27 objective. In this study, we called the ten somites from the end of the yolk extension as somites a-j, respectively, and area of columns and vessels were quantified with Zen 2 (blue edition) software. Cross-sectional images were taken at the center of each somite. For confocal time-lapse imaging, images were collected every 20 or 30 minutes for 4–6 hours.

#### Evaluation of CVP constriction

Embryos fixed in soft agar were injected with OMPT (500 or 1,000 μM) dissolved in 0.1% BSA/PBS containing 0.2% Evans blue in a total volume of 1 nl using a micro-injector in the vicinity of the heart. Time-lapse images were promptly acquired by confocal microscopy every 5 or 20 seconds for 5 minutes. For evaluation of vasoconstriction, changes in the long axis of the elliptical vessel lumen of the CVP were assessed for 5 minutes, and the rate of constriction was calculated by dividing the value at 5 minutes by the value at time 0.

### Quantification and statistical analysis

All statistical analyses were carried out using Prism software (GraphPad). Statistical significance between two groups is evaluated by Student’s t test, and p < 0.05 was considered to be significant. All data were expressed as means ± SD using Prism software (GraphPad). Zen 2 (blue edition) software was used for image quantification of vascular images. Number of samples are included in figure legends.

## Data Availability

• All data reported in this paper will be shared by the lead contact upon request. • This paper does not report original code. • Any additional information required to reanalyze the data reported in this paper is available from the lead contact upon request.
