# Supplementary material for: An ATX-LPA6-Gα13-ROCK axis shapes and maintains caudal vein plexus in zebrafish
Source: iScience. 2021 Oct 12;24(11):103254. doi: 10.1016/j.isci.2021.103254 (PMC8564058; doi:10.1016/j.isci.2021.103254)
Supplement: Document S1. Figures S1–S13 and Table S1 [file mmc1.pdf]

iScience, Volume *24*

Supplemental information

An ATX-LPA$_6$-Gα_{13}-ROCK axis shapes and maintains caudal vein plexus in zebrafish

Ryohei Okasato, Kuniyuki Kano, Ryoji Kise, Asuka Inoue, Shigetomo Fukuhara, and Junken Aoki

Supplementary Table

Table S1. List of primers used in this study, Related to STAR Methods

PCR primers used for preparing template DNA of sgRNA for transcription

Primer name	Sequence (5' to 3')
sgRNA_F	AAAAGCACCGACTCGGTGCCACTTTTTCAAGTTGATAACGG ACTAGCCTTATTTTAACTTGCTATTTCTAGCTCTAAAAC
zATXb_sgRNA_R	TAATACGACTCACTATAGGACTCACGCTCCCAGAATGGTTT TAGAGCTAGAAATAGC
zLPA$_6$a_sgRNA_R	TAATACGACTCACTATAGGTGTTTAGCATCGTCTTCAGTTT TAGAGCTAGAAATAGC
zLPA$_6$b_sgRNA_R	TAATACGACTCACTATAGGTCAACGCTAATGCACGTGGTTT TAGAGCTAGAAATAGC
zGα_{13}a_sgRNA_R	TAATACGACTCACTATAGGTACTCGCTGGATGACACTGTTT TAGAGCTAGAAATAGC
zGα_{13}b_sgRNA_R	TAATACGACTCACTATAGGACGGGGATGACTTCGATAGTTT TAGAGCTAGAAATAGC

PCR primers used for detecting mutation

Primer name	Sequence (5' to 3')
zATXb_F	TCATGTGGAACTCACGCTCCC
zATXb_R	CAGTGTGTAGAGGTTGGGGAAG
zLPA$_6$a_F	GCTCAATGTGAGCAACGTCA
zLPA$_6$a_R	AGATGTACATGGCGGCAACA
zLPA$_6$b_F	AACATGTACGGCAGCATCCTCT
zLPA$_6$b_R	CCGGACAGAAGAACCACCCAG
zGα_{13}a_F	GCGATGGTTCGAGTGCTTCG
zGα_{13}a_R	GACTCCGTGAGCCTGTTGGT
zGα_{13}b_F	CCACTTTCCTCAAGCAGATGCG
zGα_{13}b_R	TTGGCCCCTCGCTAGTCTTC
zATXa_F	TTGTAGGATCCTGTGGAACAC
zATXa_R	AAATCTGCAATTAATACATACCGTT
zLPA$_4$_F	TTGTCTACGCAATGGTACGC
zLPA$_4$_R	CCACCGGGTCAAAGAAGCAA

Supplementary Figure

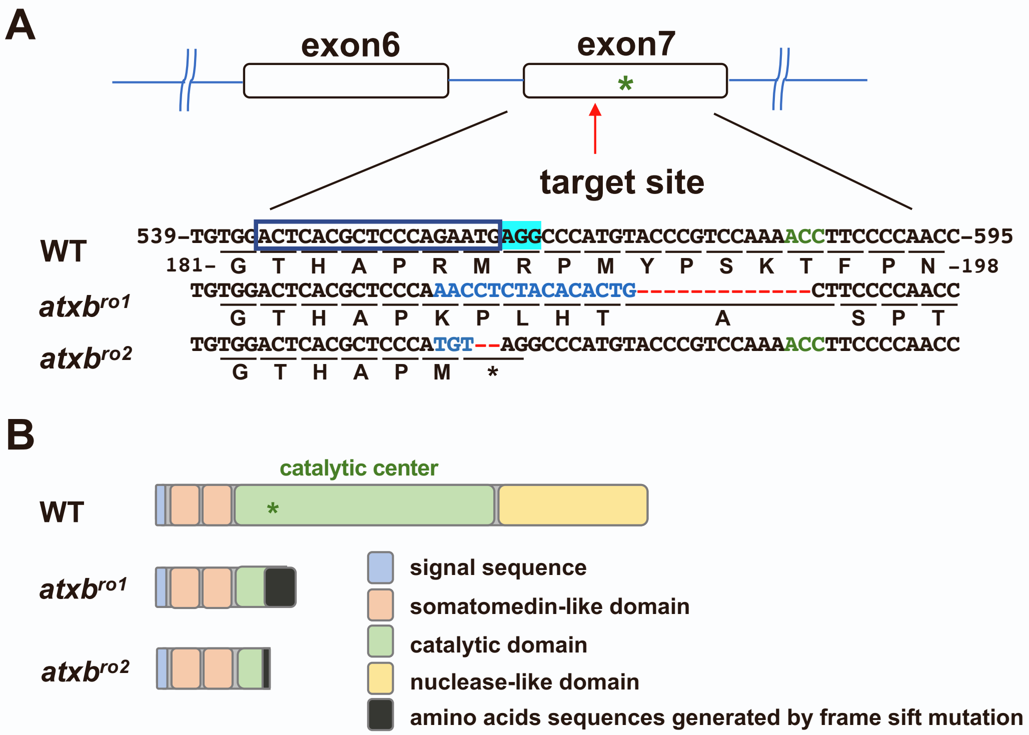

Figure S1. Generation and genomic characterization of *atxb* mutant zebrafish, Related to Figure 1
(A) The above diagram shows a schematic representation of the genomic structure and nucleotide and amino acid sequences of *atxb* gene around the target site. The nucleotide and amino acid sequences of two mutants produced using the CRISPR/Cas9 system are also shown. The location of codon sequence coding for the catalytic center is shown in asterisk, which corresponds to the threonin195. We targeted nucleotides a little upstream on the 5' side of the catalytic center for efficient gene disruption. The sgRNA target sequences are boxed, and the three nucleotides shadowed in magenta point the PAM sequence—the three nucleotides in light green code for threonin195, the catalytic center. The deleted bases in the mutant alleles are indicated by red bars. The inserted bases are shown in blue letters. (B) The figure shows domain structures of an intact ATXb protein and the predicted domain structures of mutant ATXb proteins deduced from the nucleotide sequences of the two *atxb* mutants in (A). The catalytic center is shown by an asterisk. Both mutant ATX proteins lack the catalytic center and the following domain structures due to frameshift mutations.

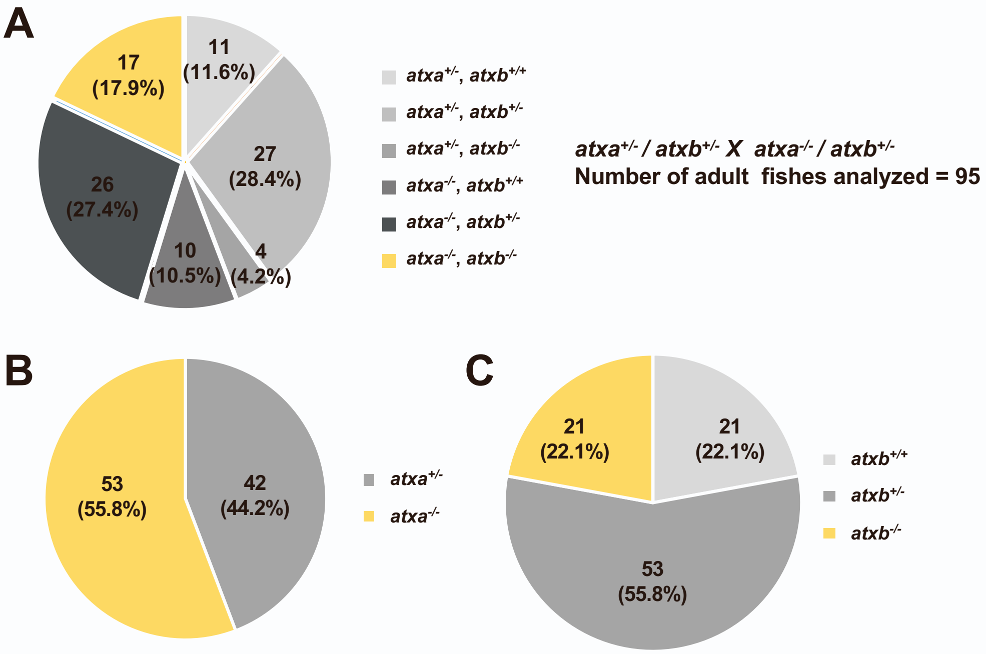

Figure S2. The ratio of each genotype of adult fishes obtained by crossing *atxa/atxb* mutants, Related to Figure 1

(A-C) *atxa*$^{+/-}$/*atxb*$^{+/-}$ and *atxa*$^{-/-}$/*atxb*$^{+/-}$ (*ro1* line) were intercrossed, and the genotypes of the resulting adult fishes were determined. Along with the number of adult fishes with each genotype, the ratio of each genotype is shown in the brackets. Graphs created by focusing on the genotype of *atxa* (B) or *atxb* (C) are also shown.

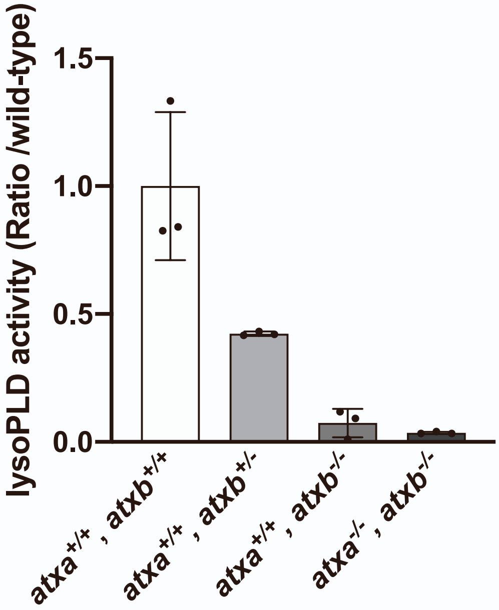

Figure S3. Biochemical characterization *atxa/atxb* mutant fish, Related to Figure 1

ATX (lysoPLD) activity of plasma from adult fish with each genotype (*wild-type* (*atxb*$^{+/+}$/*atxb*$^{+/+}$), *atxa*$^{+/+}$/*atxb*$^{+/-}$, *atxa*$^{+/+}$/*atxb*$^{-/-}$, and *atxa*$^{-/-}$/*atxb*$^{-/-}$). Plasma from *atxb*$^{-/-}$ loses most of the lysoPLD activity. LysoPLD activity was expressed by relative activity with the activity of *wild-type* (*atxa*$^{+/+}$/*atxb*$^{+/+}$) being 1. Numbers of fish analyzed are; *atxa*$^{+/+}$/*atxb*$^{+/+}$ n=3, *atxa*$^{+/+}$/*atxb*$^{+/-}$ n=3, *atxa*$^{+/+}$/*atxb*$^{+/-}$ n=3, *atxa*$^{-/-}$/*atxb*$^{-/-}$ n=3. The data were expressed as mean with SD.

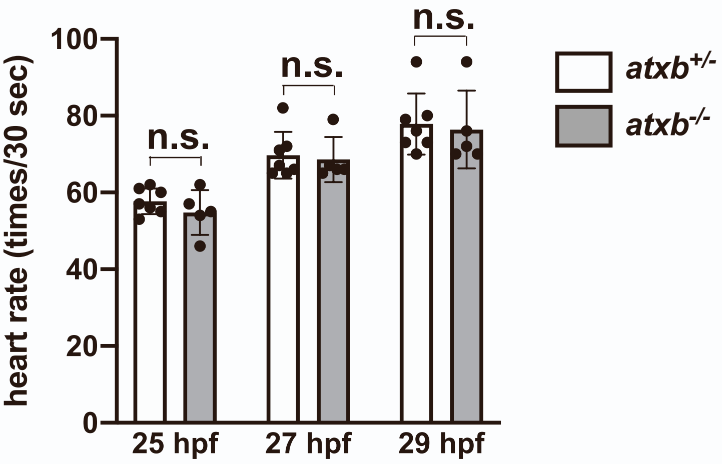

Figure S4. Heart rate is normal in ATXb KO embryos, Related to Figure 1
Heart rate of ATXb hetero mutant (*atxb*$^{+/-}$) and ATXb homo mutant (*atxb*$^{-/-}$) embryos were counted at the indicated time points, respectively. Data were shown as mean with SD of seven *atxb*$^{+/-}$ and five *atxb*$^{-/-}$ embryos. P-value was calculated by the student's t-test (n.s., no significance).

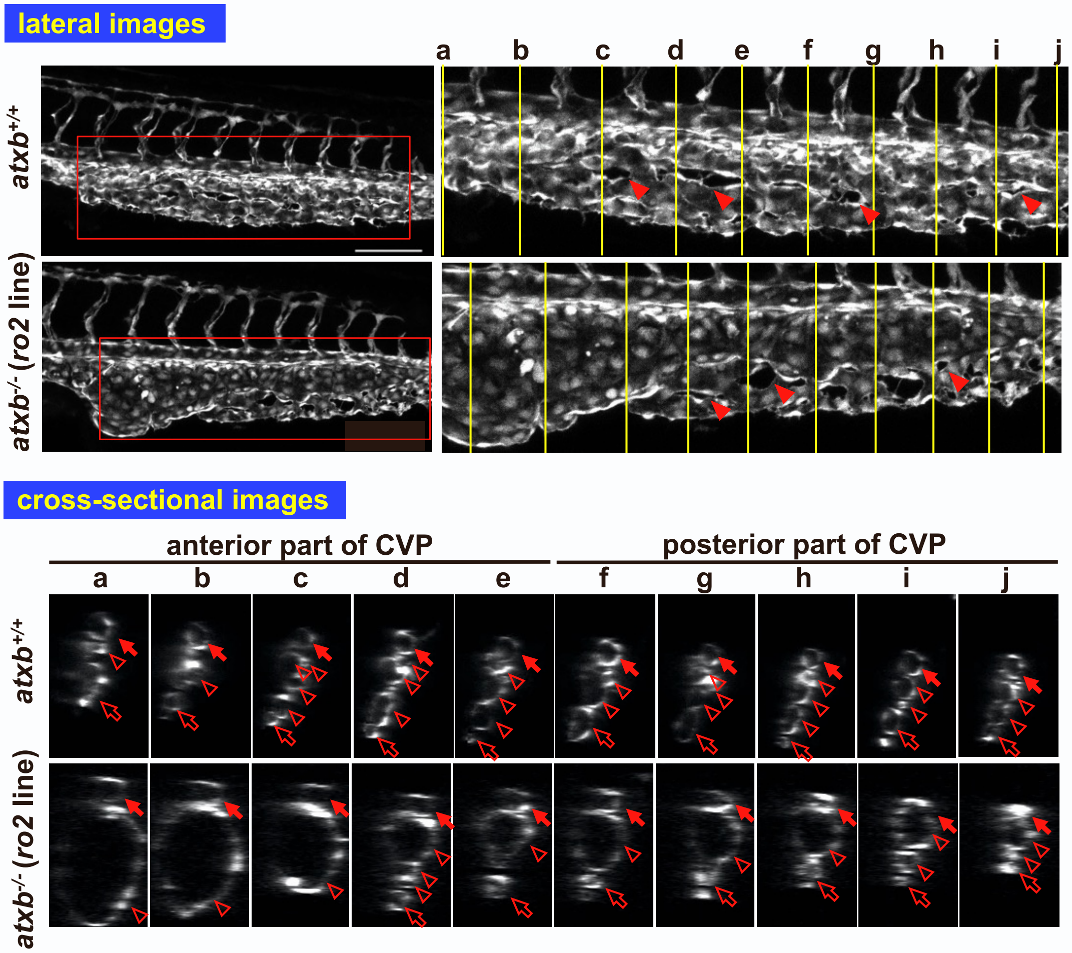

Figure S5. Similar abnormal CVP structure in *atxb* mutant (*ro2* line) embryos, Related to Figure 1
Projection views of confocal z stack images and cross-sectional images of CVP of wild-type and *atxb* KO (*ro2* line) embryos at 36 hpf. Enlarged images of the area surrounded by squares are positioned in the right side (upper panel). The cross-sectional images at the positions indicated by yellow lines in the upper projection view are shown in the lower column. Ten somites from the end of the yolk extension were analyzed. Somites a to e and somites f to j were defined as anterior and posterior somites, respectively. Arrowheads indicate the column structures. Arrows, hollow arrowheads and hollow arrows in the lower panels indicate the CA, dCVP and vCVP, respectively. Essentially, the same phenotypes as in the *ro1* line were observed, including the non-disjunction of CA and dCVP. Scale bars, 100 µm

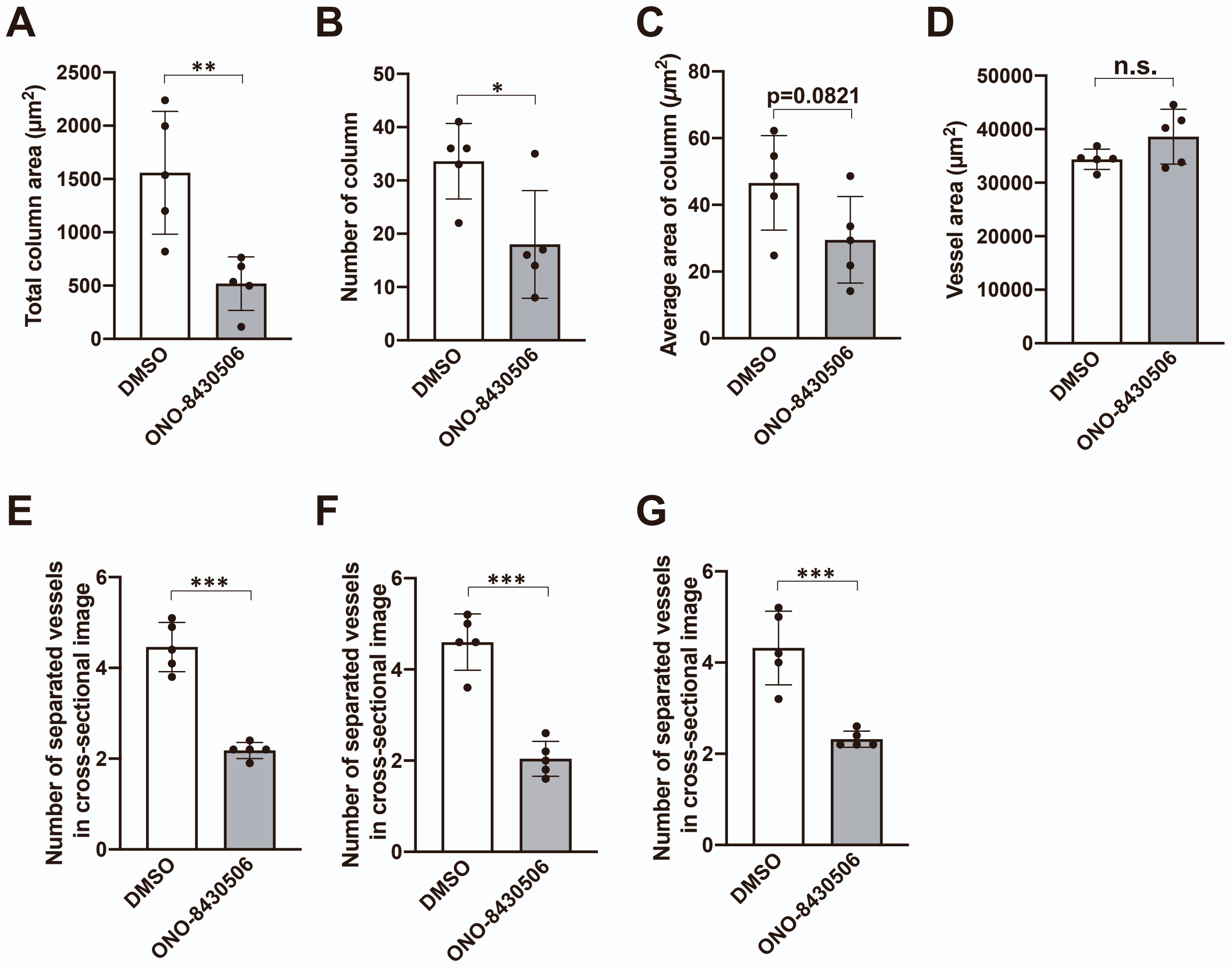

Figure S6. Abnormal caudal vein plexus (CVP) structure in embryos treated with ATX inhibitor, Related to Figure 1

(A-G) Quantitative evaluation of CVP's morphology from confocal images. Wild-type embryos were treated with ATX inhibitor, ONO-8430506, from 25 hpf to 36 hpf. Confocal images have acquired at 36 hpf. Ten somites from the end of the yolk extension were evaluated. Data were shown as means ± SD of five embryos treated with DMSO, and five embryos treated with ONO-8430506. P-value was calculated by the student's t-test (*p<0.05; **p<0.01; n.s., no significance). (A) The total area of columns in the ten somites was quantified by Zen 2 (blue edition) software and shown. (B) The total number of columns present across the ten somites. (C) The average area of columns (Total area of columns (A) divided by the number of columns (B)). (D) Total vessel area. EGFP-positive area was quantified as a vessel area. (E-G) The graphs show the average number of separated vessels in the cross-sectional images from ten somites (somites a to j, E), five anterior somites (somites a to e, F), and five posterior somites (somites f to j, J). The numbers of embryos analyzed are indicated.

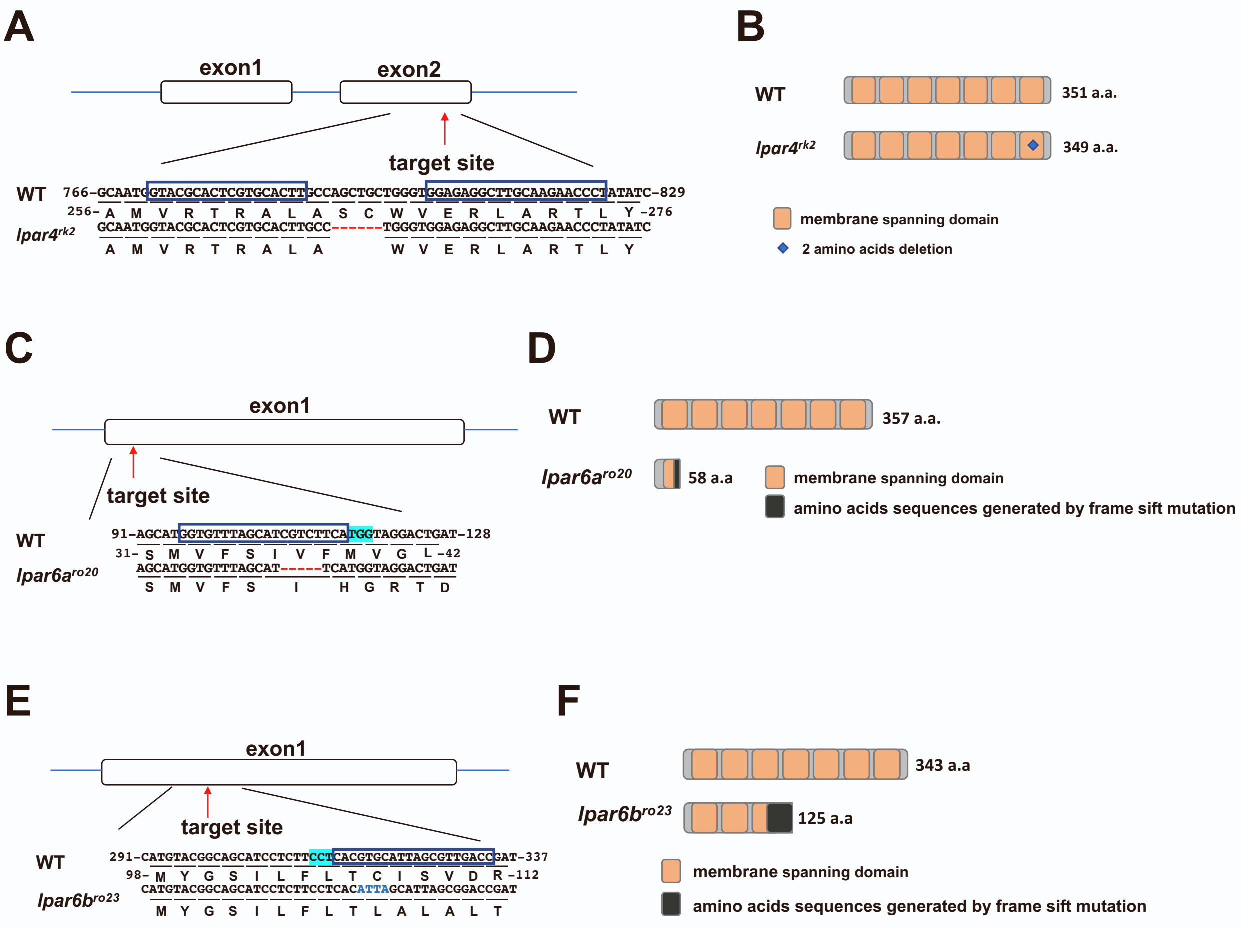

Figure S7. Generation and genomic characterization of LPA receptor (*lpar4, lpar6a, lpar6b*) mutant fish, Related to Figure 3

(A, C and E) Schematic representation of the genomic structure and sequence of mutations induced by genome editing technology. (B, D and F) Domain structures of an intact protein and the predicted domain structures of mutant proteins deduced from the nucleotide sequences as shown in Supplementary Fig. 1. (A and B) *lpar4* mutant was generated by using TALEN. The TALEN target sequences are boxed. (C and D) *lpar6a* mutant was generated by using the CRISPR system. (E and F) *lpar6b* mutant was also generated by using the CRISPR system as shown in Supplementary Fig. 1.

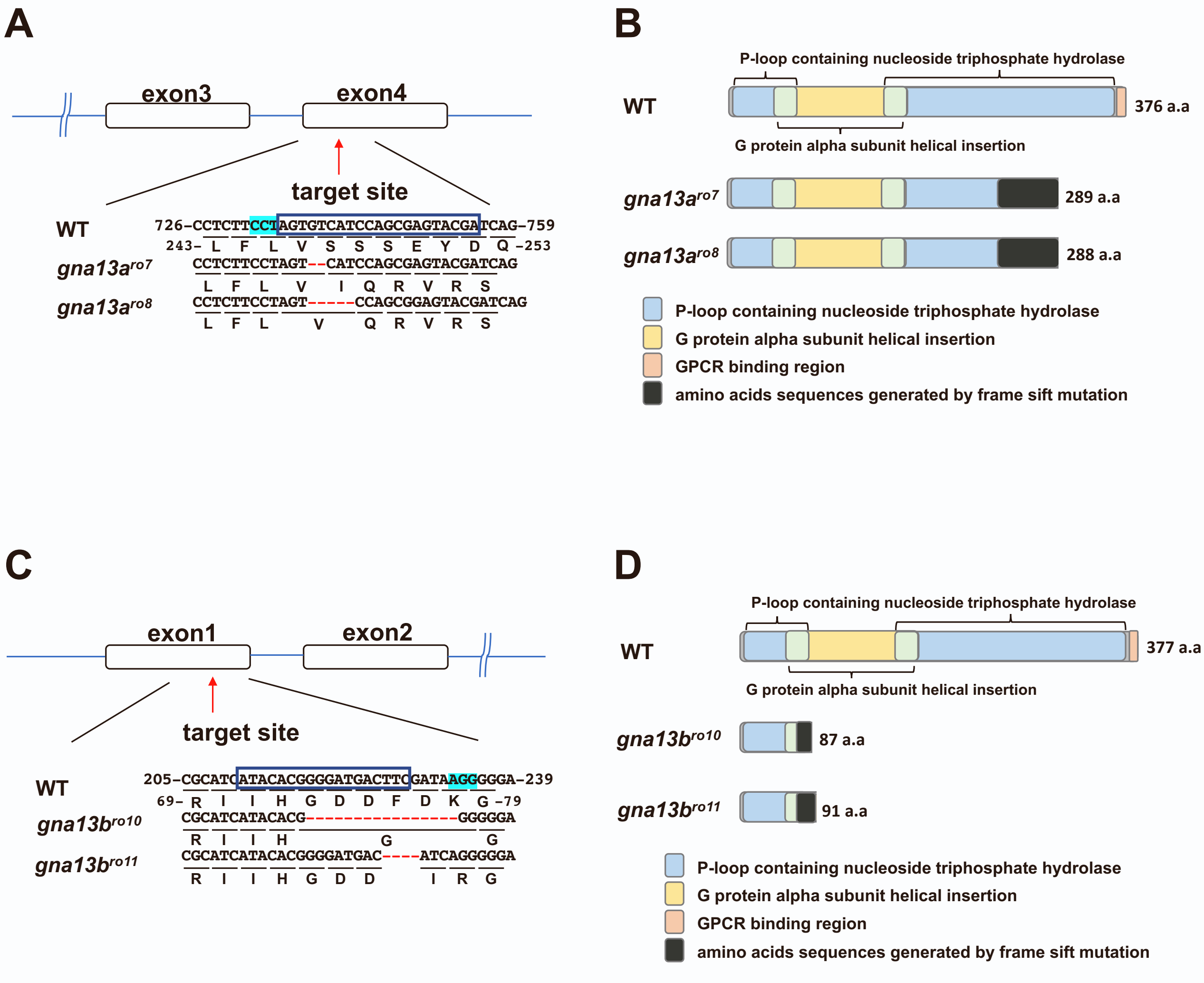

Figure S8. Generation and genomic characterization of Gα_{13} (*gna13a, gna13b*) mutant fish, Related to Figure 4

(A and C) Schematic representation of the genomic structure and sequence of mutations induced by genome editing technology. (B and D) Domain structures of an intact protein and the predicted domain structures of mutant proteins deduced from the nucleotide sequences as shown in Supplementary Fig. 1. Gα_{13}a and Gα_{13}b protein express from *gna13a*^{ro7} and *gna13a*^{ro8} respectively lose the functional part which is necessary for binding with GPCR.

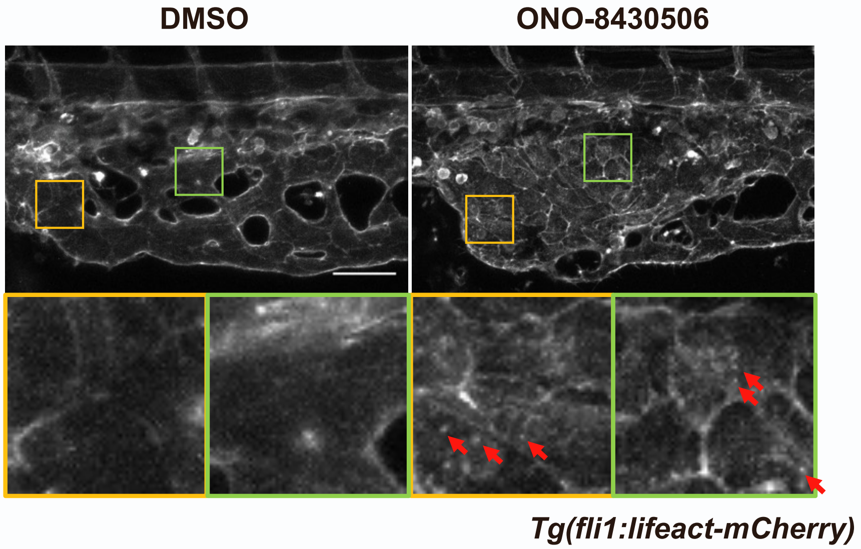

Figure S9. Administration of ATX inhibitor ONO-8430506 affects actin cytoskeleton in CVP, Related to Figure 4

Tg(fli1:lifeact-mCherry) embryos were treated with ONO-8430506 ATX inhibitor at 25 hpf. Shown are projection views of confocal z stack images of CVP from the lateral side of *Tg(fli1:lifeact-mCherry)* embryos at 36 hpf. Enlarged images of the areas surrounded by squares are positioned in the lower side. Note that punctate signals are significantly increased in embryos treated with ATX inhibitor. Arrows indicate the punctate signals. Scale bars, 50 µm.

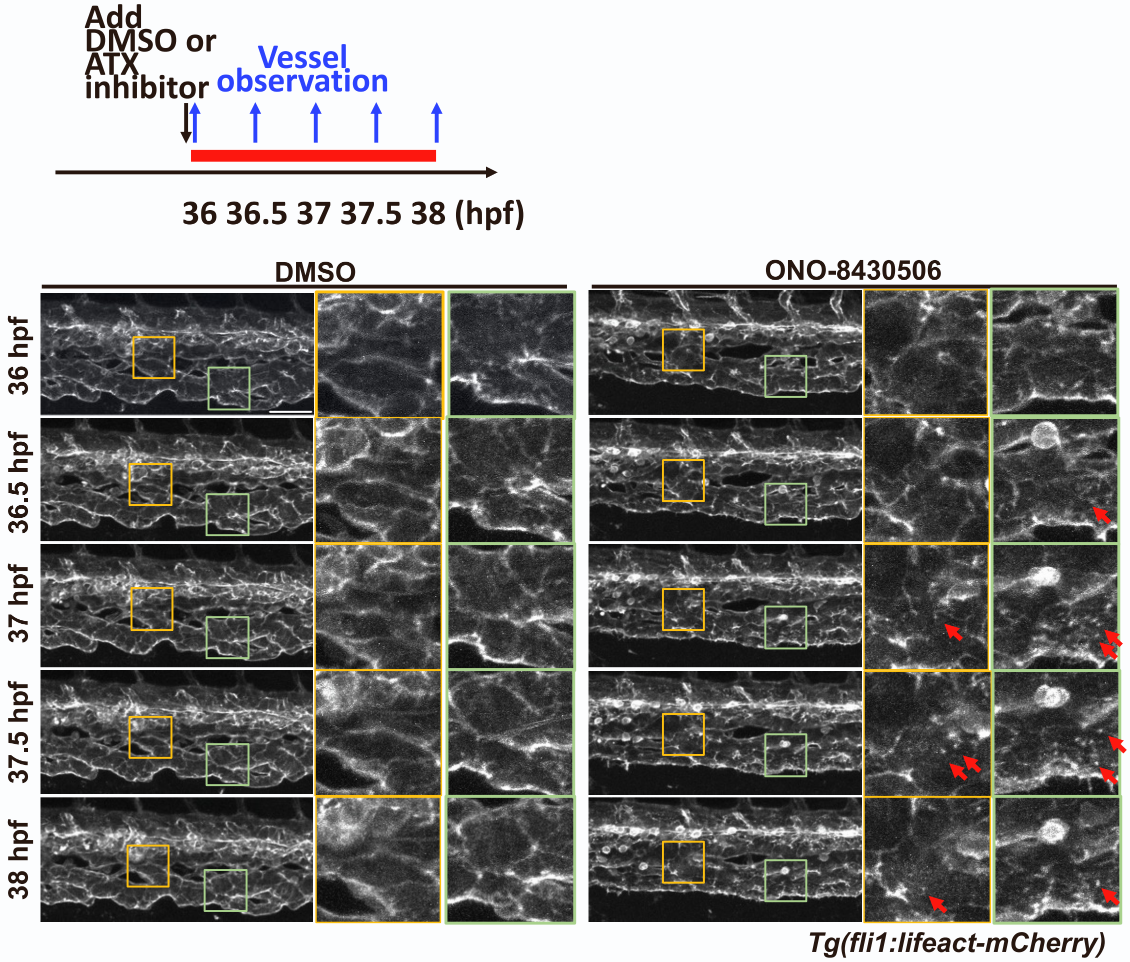

Figure S10. Observation of the actin cytoskeleton in ATX inhibitor-induced CVP column regression, Related to Figure 5

Tg(fli1:lifeact-mCherry) embryos were treated with ONO-8430506 ATX inhibitor at 36 hpf and sequential time-lapse images were acquired at the indicated time points. Schematic diagrams of the protocol are shown in the upper side. Enlarged images of the area surrounded by squares are shown in the right side. Note that punctate signals are significantly increased in embryos treated with ATX inhibitor 30 minutes after the treatment. Arrows indicate the punctate signals. Scale bars, 50 µm.

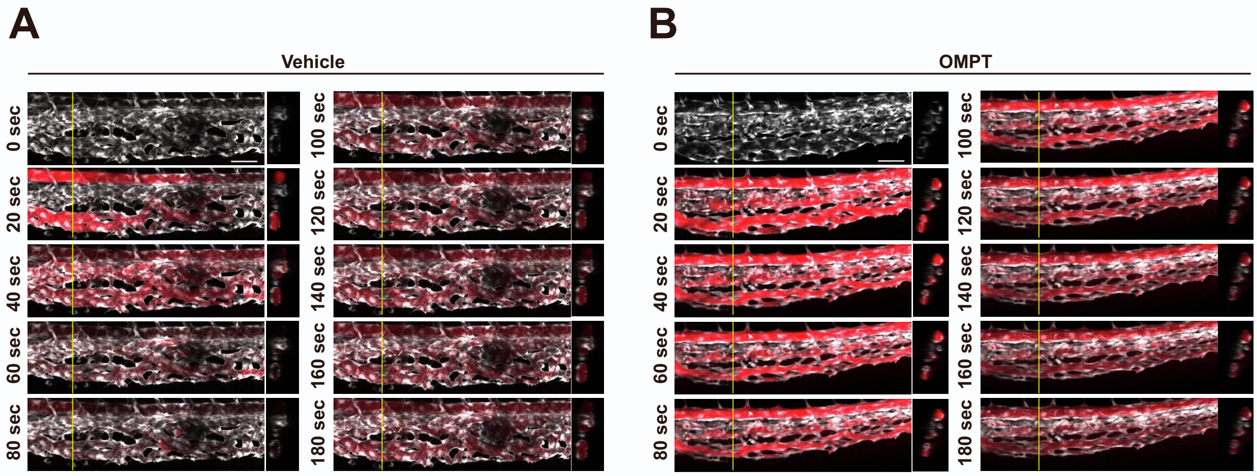

Figure S11. Constriction of CA and CVP by an LPA stable analog, Related to Figure 6

(A and B) Constriction of CVP induced by OMPT. At 36 hpf, the embryos were injected with OMPT in the vicinity of the heart. Time-lapse images are taken every 20 seconds after the injection. The circulation of OMPT is evaluated by the fluoresce of Evans Blue, which is mixed with OMPT. OMPT rapidly induces shrinkage of CVP as soon as it reaches CVP (B), which is never observed in vehicle control (DMSO, A). OMPT also induces the contraction of CA (B). Scale bars, 50 µm.

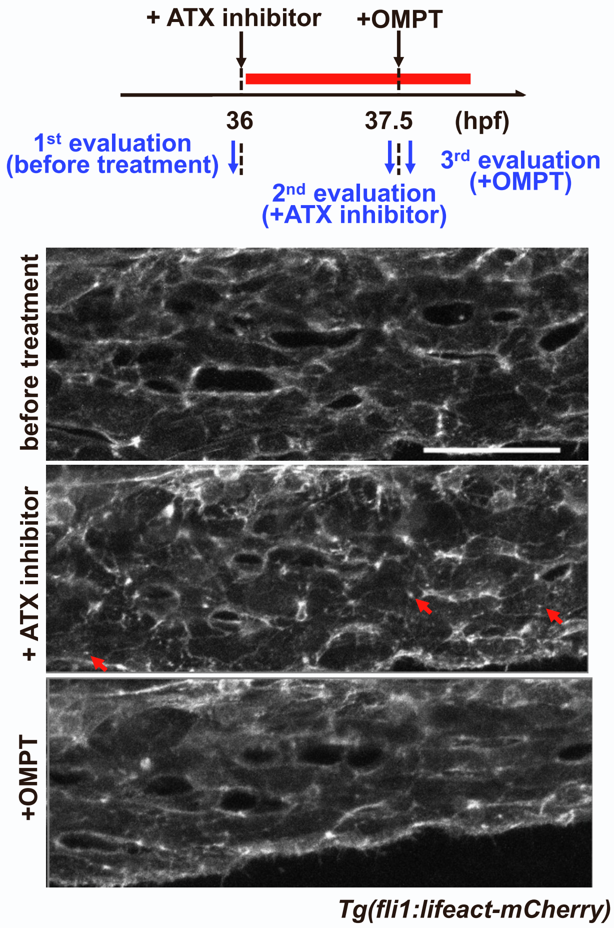

Figure S12. Observation of actin cytoskeleton in CVP constriction induced by LPA stable analog, Related to Figure 6

Projection view of confocal z stack images of CVP from the lateral side of *Tg(fli1:lifeact-mCherry)* embryos. Images were taken at 36 hpf just before ATX inhibitor treatment (upper), at 37.5 hpf 1.5 hour after the treatment (middle), and at 37.6 hpf just after the injection of OMPT (lower). Arrows indicate the punctate signals. Scale bars, 50 µm.

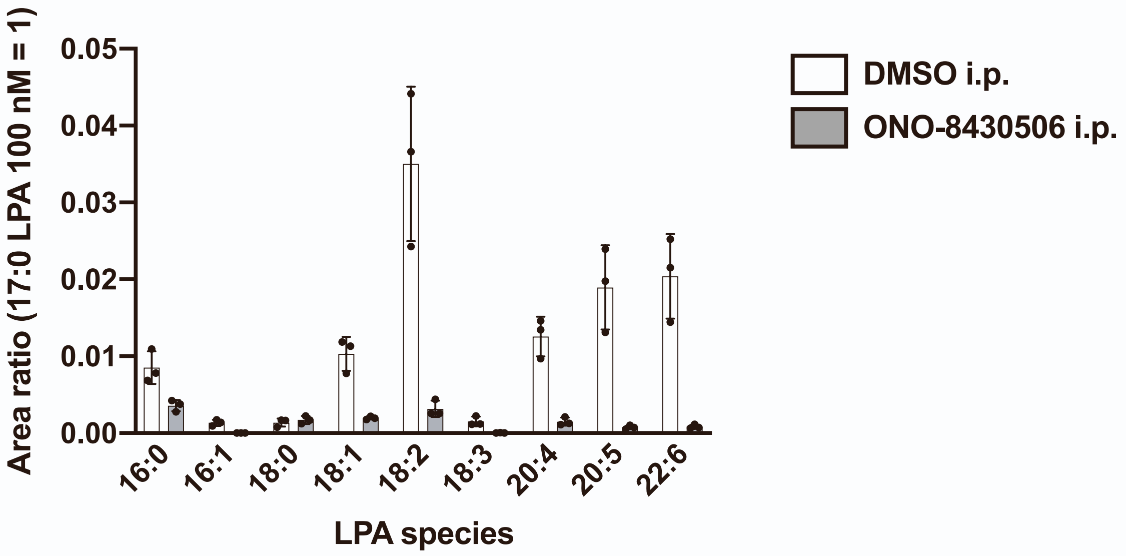

Figure S13. Measurement of plasma LPA levels in adult fishes treated with ATX inhibitor, Related to Figure 1

Adult fishes were treated with DMSO or ATX inhibitor by intraperitoneal injection. LPA levels of plasma was measured by LC-MS/MS (DMSO n=3, ONO-8430506 n=3). The data were expressed as mean with SD.
